# Differences in Visual-Spatial Input May Underlie Different Compression Properties of Firing Fields for Grid Cell Modules in Medial Entorhinal Cortex

**DOI:** 10.1371/journal.pcbi.1004596

**Published:** 2015-11-19

**Authors:** Florian Raudies, Michael E. Hasselmo

**Affiliations:** 1 Center for Systems Neuroscience and Department of Psychological and Brain Sciences, Boston University, Boston, Massachusetts, United States of America; 2 Center for Computational Neuroscience and Neural Technology, Boston University, Boston, Massachusetts, United States of America; University College London, UNITED KINGDOM

## Abstract

Firing fields of grid cells in medial entorhinal cortex show compression or expansion after manipulations of the location of environmental barriers. This compression or expansion could be selective for individual grid cell modules with particular properties of spatial scaling. We present a model for differences in the response of modules to barrier location that arise from different mechanisms for the influence of visual features on the computation of location that drives grid cell firing patterns. These differences could arise from differences in the position of visual features within the visual field. When location was computed from the movement of visual features on the ground plane (optic flow) in the ventral visual field, this resulted in grid cell spatial firing that was not sensitive to barrier location in modules modeled with small spacing between grid cell firing fields. In contrast, when location was computed from static visual features on walls of barriers, i.e. in the more dorsal visual field, this resulted in grid cell spatial firing that compressed or expanded based on the barrier locations in modules modeled with large spacing between grid cell firing fields. This indicates that different grid cell modules might have differential properties for computing location based on visual cues, or the spatial radius of sensitivity to visual cues might differ between modules.

## Author Summary

How do we navigate from one location to another and how do we represent space to accomplish this task? Researchers have collected data from the entorhinal cortex in rodents to answer these questions, finding grid cells that fire whenever a rodent traverses through an array of locations falling on the vertices of tightly packed equilateral triangles. Grid cells with large spacing (large side lengths of the triangles between firing fields) are distorted when the environment is manipulated, e.g. by pushing walls or inserting walls in a box. In contrast, grid cells of small spacing remain largely unaffected by such manipulations. We present a computational model to explain this behavior of grid cells. In our model information about the motion of features on the ground, which are unaffected by wall manipulations, drive grid cells with small spacing between firing fields, while static features like landmarks, which are affected by wall manipulations, drive grid cells with large spacing between firing fields. These differences could correspond to different positions within the visual field of the animal. This model puts forth a testable hypothesis about the type of features that drive grid cells of different spacing.

## Introduction

The generation of action potentials by grid cells in the rat medial entorhinal cortex depends upon the location of the rat as it forages in an open field environment [1–3]. Grid cells fire when a rat visits a regular array of locations falling on the vertices of tightly packed equilateral triangles. This regular pattern of firing has been proposed to partly arise from the path integration of self-motion cues along the rats trajectory [4–6]. However, experiments have also demonstrated a clear dependence of grid cell firing on the location of sensory cues in the environment. For example, grid cell firing fields rotate with the location of a white cue card on the cylindrical wall of an environment [2], as previously shown for place cells [7].

The dependence of grid cell firing on sensory cues was clearly demonstrated by a further manipulation in which the barriers of an open field environment were systematically shifted between trials [8]. For example, when an environment was altered from a square with sides of 100 cm to a rectangle with sides of 70 cm and 100 cm, the grid cell firing fields showed compression of their spacing primarily in the dimension of the compression of the barriers [8]. This did not require regular physical contact with the walls of the environment (e.g. with the whiskers), suggesting sensitivity to the visual cues of the barriers.

Later research demonstrated that this compression of the spacing of grid cell firing fields could be selective for individual populations of grid cells within medial entorhinal cortex [9]. This study demonstrated that separate populations, or modules, of grid cells in medial entorhinal cortex shared orientation and spacing, and demonstrated up to five modules with different spacing [9]. The same study also demonstrated that compression of the barriers of the environment could selectively affect modules with larger spacing between grid cell firing fields (usually recorded from more ventral regions of the medial entorhinal cortex) while having no effect on modules with narrower spacing between firing fields (usually recorded from more dorsal regions of the medial entorhinal cortex).

The selective effect on individual modules suggests differences in the mechanism of influence of visual features on grid cell modules, and also indicates that these mechanisms of spatial location do not involve only path integration based on vestibular and proprioceptive input. The influence of distant visual features on barriers indicates that the sensory input processed by visual cortices on the dorsal surface of the rat brain [10–14] can influence the spatial computations in the medial entorhinal cortex. Data indicates that rodent visual cortices might have separate systems sensitive to differences in speed of movement and spatial frequency of stimuli [11–13] that may resemble the dorsal and ventral streams observed in primate cortices [15]. There is also evidence that different rodent visual regions are restricted to specific portions of visual space [14] and may project selectively to specific entorhinal regions [12].

In a nutshell, our computational model employs two feature systems: A moving feature system and a static feature system. These are both abstract analytical models of the processing of visual stimuli that provide input to network models of grid cells. The moving feature system uses optic flow, which describes the movement of features on the rat’s retina, which is caused by the relative motion between the rat and the environment. This optic flow is then used to estimate the self-motion of the rat. A temporal integration of this velocity signal can generate grid cell firing, e.g. using the velocity controlled oscillator model [6, 16, 17]. The static feature system uses sensed, visual angles of landmarks and the memorized location for these landmarks to triangulate the current location of self. The triangulation method computes the intersection point of all observed visual angles (slope) together with their memorized location (offset) using a least-squares optimization based on the deviation of intersecting points and the solution. If the environment has changed since the last time of memorization of landmark locations, a conflict appears between the sensed and memorized signal. In our model, we resolve this conflict by introducing a compression of the distance metric. For this reason the environment and the model grid cells in the static feature system appear compressed.

**Fig 1:**
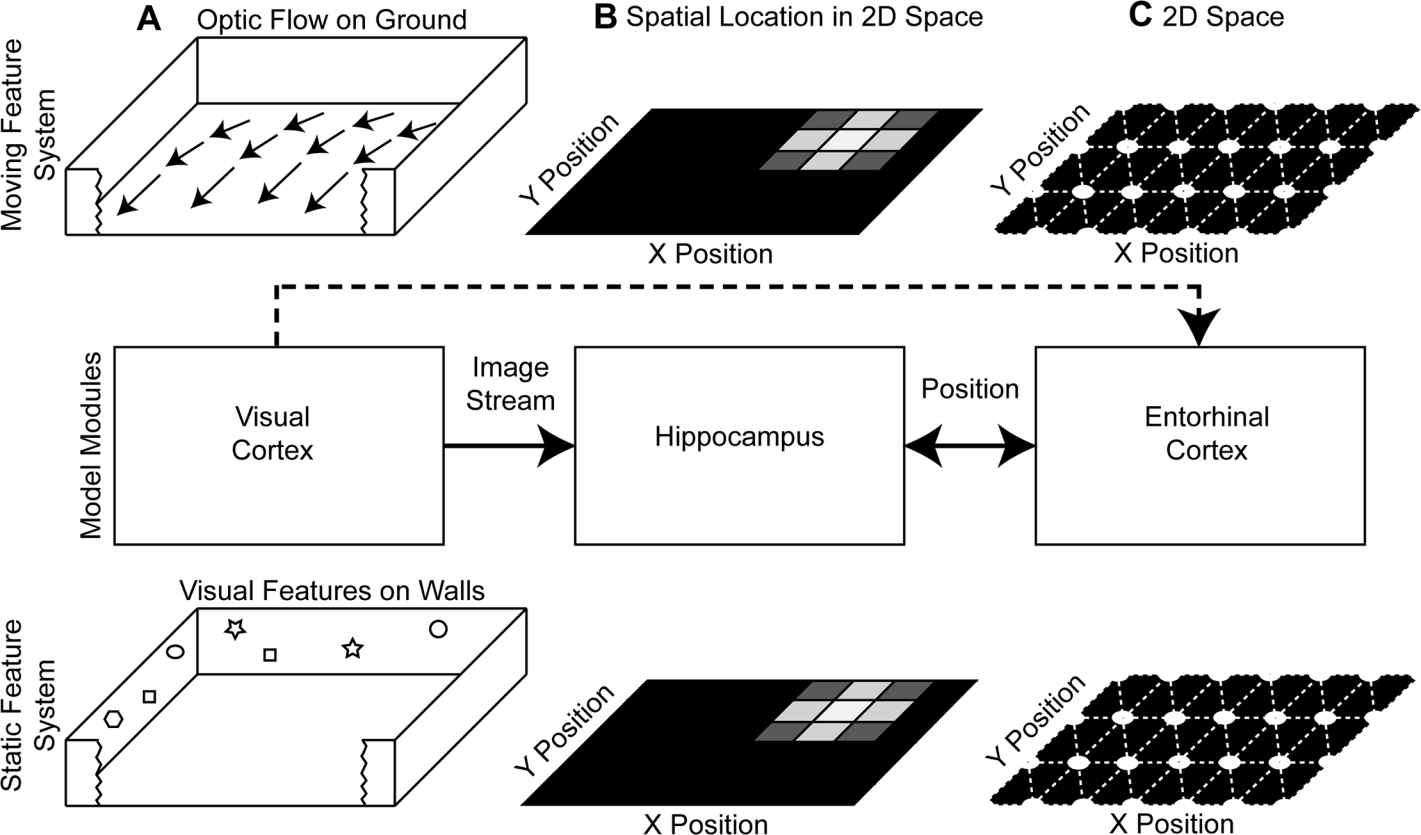
Shows the model components. (A) The visual cortex and other cortical areas provide an image stream with optic flow and visual features. (B) Processing in cortical areas provides input to the hippocampus and allows for a mapping between the sensed image stream and the representation of spatial location. (C) In our model entorhinal cortex has two modules generated by functionally different mechanisms, estimating location through optic flow for the moving feature system or through triangulation for the static feature system. In this diagram, we also included the anatomical projection from visual cortex to entorhinal cortex, which is currently not used in our model. Therefore, we show this projection as dashed line.

This work presents a conceptual and computational model for the observed differences in compression between grid cells recorded from dorsal versus ventral medial entorhinal cortex. Our conceptual model assumes that these two regions receive input from different functional systems (Fig 1). In our model the moving feature system, motivated by the dorsal stream, receives information about optic flow on the ground plane (primarily in the ventral visual field), which remains unchanged by the compression in the environment. Thus, firing of model grid cells in this moving feature system remains unchanged by the compression of the environment. In contrast, the static feature system, motivated by the ventral stream, receives input from landmarks, which change with the compression of the environment (and appear more in the dorsal visual field). Thus, the compression of the environment influences model grid cells in the static feature system.

We perform a computational study, which has two aims. The first aim is to test the behavior of this model with its moving feature system and static feature system when compressing the environment through simulations. The second aim is to compare the noise sensitivity of the moving feature system and the static feature system. We organized these simulations into two sections in the results.

## Results

**Fig 2:**
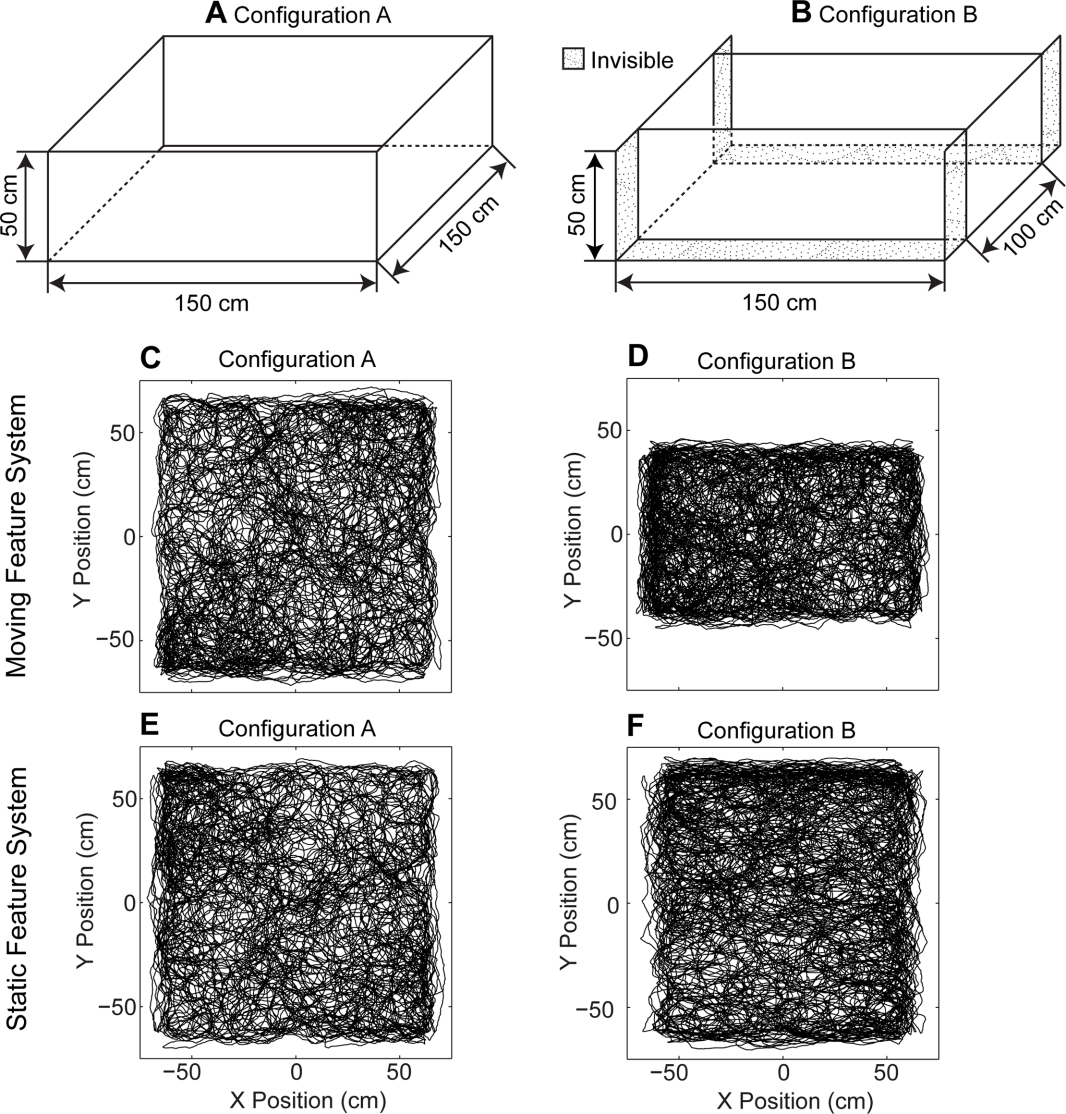
Shows compression influences the static feature system, while the moving feature system remains unaffected. The box in configuration A (part A of Fig 2) is square and for configuration B (part B of Fig 2) the box is rectangular. (C) Estimated location for the moving feature system in configuration A. (D) Estimated location for the moving feature system in configuration B showing that estimates of location correctly change with compression. (E) Estimated location for the static feature system in configuration A. (F) Estimated location for the static feature system in configuration B. This shows that this static feature system inaccurately estimates location as if the box were to appear as uncompressed.

**Fig 3:**
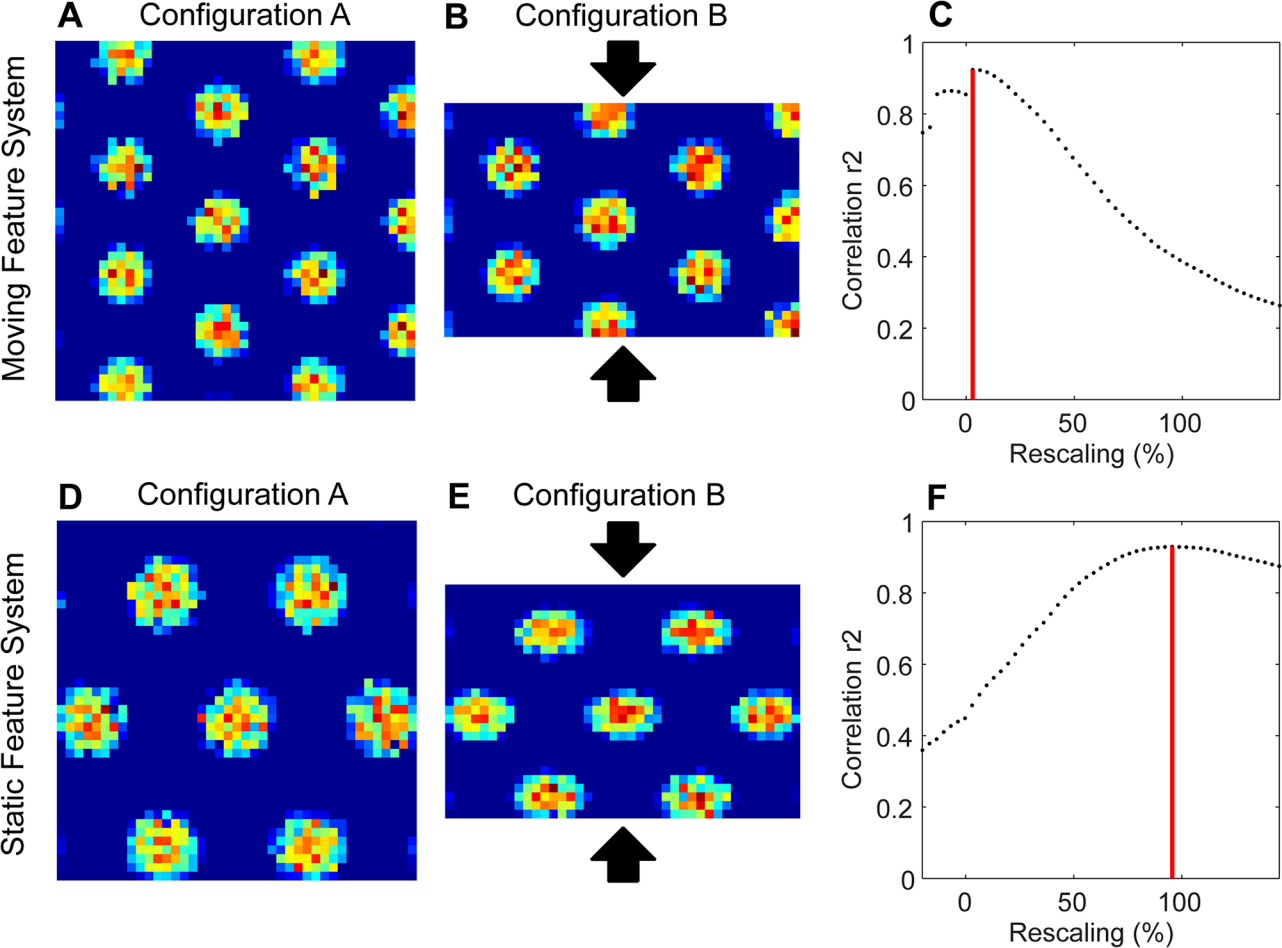
Shows the compression in configuration B compared to configuration A. (A) Simulated grid cell firing pattern from a simulated dorsal entorhinal cell for (A) configuration A (grid score 1.88) and (B) for configuration B (grid score 1.91). (C) Correlation values for different magnitudes of compression of B relative to A shows a peak correlation at a compression of ≈ 3.1%. (D) The simulated grid cell firing pattern from the ventral entorhinal cell for (D) configuration A (grid score 1.66) and (E) configuration B (grid score 1.25) based on inaccurately estimated locations in Fig 2F. (F) Correlation value for different magnitudes of compression of configuration B relative to configuration A shows that the grid cell firing in configuration B needs to be increased by a compression factor of ≈ 95.5% (about 50 cm) to obtain maximum correlation with the grid cell firing pattern from configuration A.

**Fig 4:**
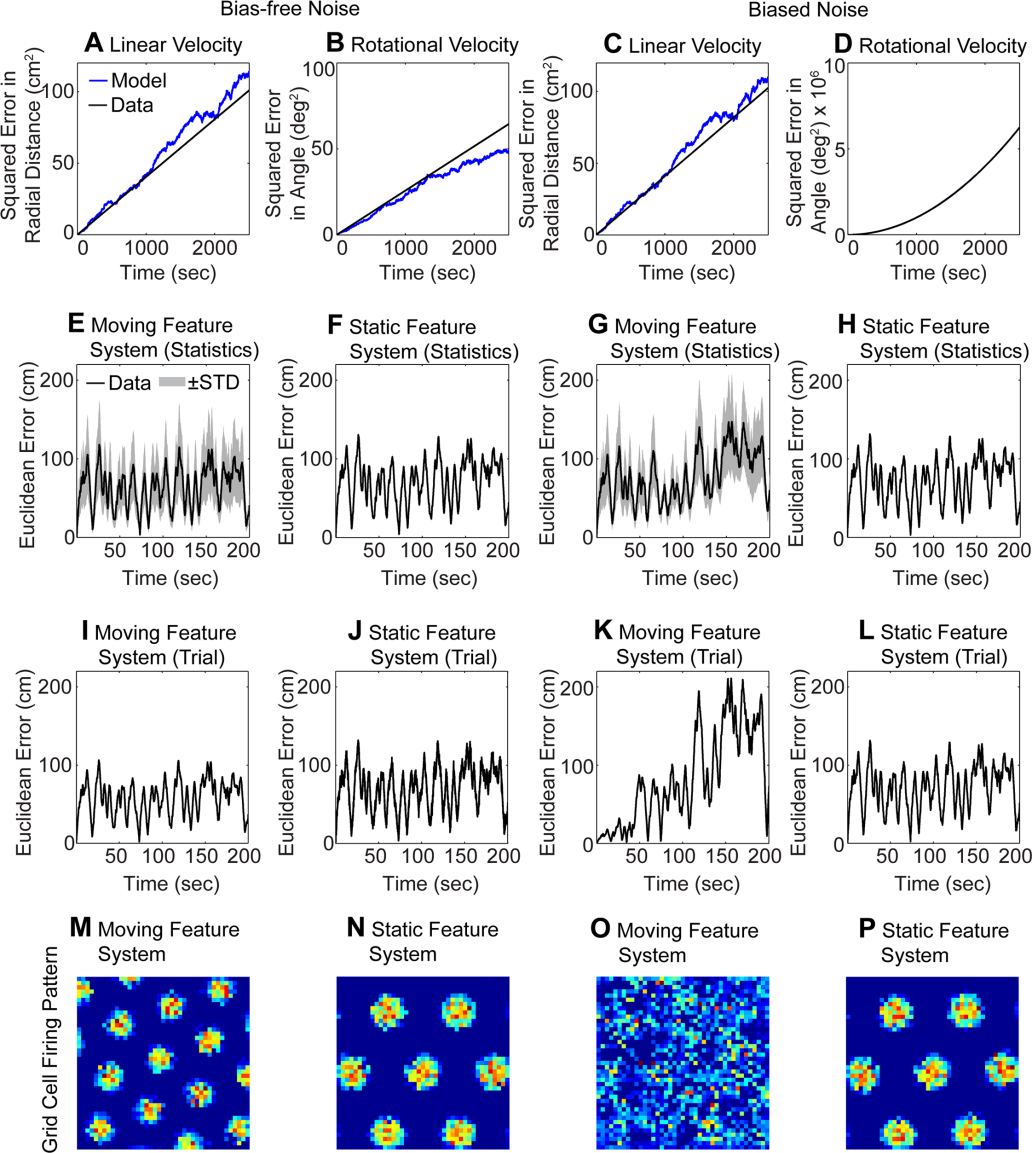
Illustrates the influence of bias-free and biased Gaussian noise on the location estimates and grid cell firing. (A-D) shows the temporal integration without a transform from polar to Cartesian coordinates (Eq 9). Here the of estimated radial distance and angle based on the integrated linear velocity and rotational velocity follows a Brownian motion model for the bias-free noise and biased noise. (A) Squared distance error for the linear velocity for bias-free noise and (B) squared angular error for the rotational velocity for bias-free noise. (C) Squared distance error and (D) and squared angular error for biased noise. The legend in (A) applies to all panels (A-D). In (D) the blue (model) and black line (data) overlap. (E-F) Euclidean error computed from integration of position in Cartesian coordinates (Eq 9). The Euclidean error between the estimated and ground-truth location for bias-free noise is small in both the moving feature system (E) and in the static feature system (F). (G-H) For biased noise the error increases rapidly due to the error accumulation in the moving feature system (G), but the error stays almost constant for biased-free noise in the static feature system because no error accumulation happens (H). For panels (E-H) the statistics includes 100 trials, where the black line shows the mean and the shaded gray area +-1 STD. Panels (I-L) are analogous to panels (E-H) but provide data for a single trial. (M-P) The corresponding firing patterns of that single trial from the velocity controlled oscillator model are shown for: (M) bias-free noise and the moving feature system; (N) bias-free noise and the static feature system; (O) biased noise and the moving feature system; and (P) biased noise and the static feature system.

In the first simulation, we altered the configuration of a box by shifting two barriers, which altered the box from a square to a rectangular layout (Figs 2 and 3). In the second simulation, we tested the accuracy of the location estimation methods with noisy perturbations in the angle of landmarks and the angular velocities of optic flow components (Fig 4).

### Grid cell firing of the static feature system compresses while grid cell firing of the moving feature system remains unchanged for a compressed box configuration

In this simulation, we modeled the experiment on the compression of a box [8, 9]. The same box appears in two configurations. In configuration A the box is 150 cm × 150 cm (Fig 2A) and in configuration B the box is 150 cm × 100 cm (Fig 2B). This shift means that the visual features on the two shifted barriers have changed their absolute location in 3D space, here in the y-dimension of the horizontal plane. In addition, some features visible in configuration A become invisible in configuration B (dotted surfaces shown in Fig 2B). These invisible features are hidden by the shifted barriers in configuration B.

The shift of barriers has a differential effect on the different modules, dependent on the nature of the input to the different modules (Fig 2C–2F). The location estimate from the moving feature system before the change (Fig 2C) remains accurate after the alteration (Fig 2D), effectively tracking the limited range of movement in the compressed box. In contrast, the location estimates for the static feature system before the change (Fig 2E) are distorted after the box is compressed (Fig 2F), incorrectly estimating that the movements in the environment cover the same region, despite the compression. In our example the compression of the box is 33%. To visualize the grid cell response to compression and to compare this compression effect in location estimates with published experimental data on the compression effect in grid cell firing, we used the velocity controlled oscillator model [6] with our location estimates to simulate the behavior of grid cells. This visualization and analysis of compression effects does not depend on the type of grid cell model used. The results are the same when using an attractor model of grid cells [18] instead (S1 Fig).

We matched the compression of grid cell firing patterns from configuration B (Figs 3B and 2D) with grid cell firing patterns of configuration A (Figs 3A and 2C). The match of firing fields was measured by the r^2^ score. This comparison focuses on the correlation of firing fields, not the correlation of the boundaries of the environment. Across different possible compression percentages, we found a maximum match (maximum r^2^ score) at ≈ 3.1% using inputs from the moving feature system (Fig 3C). This means that there was almost no compression necessary to match the grid cell firing pattern in configuration B with the grid cell firing pattern in configuration A. The change in the x-dimension to match the two firing patterns is close to 0% for the moving feature system.

In the case of the static feature system, location estimates in configuration B are based on the memorized appearance of features in the box that are matched to the altered visual appearance of these features through compression factors. Essentially, the model estimates locations within the box in configuration B (Fig 2F) as if the walls were still in configuration A (Fig 2E). We used these location estimates within the velocity controlled oscillator model to simulate grid cell firing. The resulting grid cell firing pattern based on the actual location of the rat shows a clear compression of relative spacing of firing fields with the alteration between configuration A and configuration B (compare Fig 3E with 3D). When comparing across different percentages of compression, a maximum match in correlation between the grid cell firing patterns from configuration A (Fig 3D) and configuration B (Fig 3E) appears for the compression of ≈ 95.5% (Fig 3F). That is, the grid cell firing pattern from configuration B had to be expanded in the y-dimension by ≈ 95.5% (of the 50 cm barrier shift) to best match the grid cell firing pattern from configuration A. This indicates a substantial compression of the relative spacing of grid cell firing fields for the static features system with the change in barrier location. To match the Stensola et al. procedures [9], the percentage value for 100% compression indicates that the expansion of the environment is equal to the full amount (100% or 50 cm) to make the match of the configuration B to configuration A.

In sum, the firing recorded from these simulated grid cells using the theorized mechanism of optic flow on the ground plane in dorsal entorhinal cortex (moving feature system) remain unchanged by alterations of barriers in the box. In contrast, the firing recorded from simulated grid cells using the theorized mechanism of triangulation from visual features proposed for ventral entorhinal cortex (static feature system) shows a compression proportional to the shift of barriers. This corresponds to the measured data from grid cells in environments with barrier shifts [8, 9].

In our model this difference in compression of different cells is explained by using two functionally different mechanisms to model the location estimate for the dorsal entorhinal cells versus the ventral entorhinal cells. For the dorsal entorhinal cells location is estimated from optic flow on the ground plane (moving feature system), which remains unchanged when barriers are altered. This might indicate sensitivity to features in the ventral visual field (dorsal retina). For the ventral entorhinal cells location is estimated from landmark locations (static feature system) and those estimates change according to the shift of the walls that shift landmark location. This might indicate sensitivity to features in the more dorsal visual field (ventral retina). Our model is completely uniform in response to different walls, so we get the same results for compression in the x-dimension instead of the y-dimension. Shifting one or two walls is equivalent in our model as long as the reference (0, 0)-location is adjusted accordingly.

### Noise for velocity based position estimates accumulate error while noise does not accumulate error for triangulation-based position estimates

The moving feature system estimates position by integrating velocities over time and, thus, this moving feature system also temporally integrates any error in such velocity estimates. However, the static feature system estimates the position at each time point and, thus, does not integrate any error over time. To test, visualize, and understand the noise sensitivity of our model further, we used the same random trajectory as for the box with dimensions of 150 cm × 150 cm (configuration A). We superimposed independent, additive Gaussian noise onto the angular directions of landmarks and the angular velocities of sensed feature locations on the ground (see Methods). To compare the noise in the two different domains, we computed the Signal-to-Noise Ratio (SNR) value, which we matched between noise in angles and noise in angular velocities. We performed simulations with Gaussian noise with two parameterizations. For the first parameterization, noise in the moving feature system had a mean value of *μ*_*v*_ = 0 deg/sec and a standard deviation (STDs) of *σ*_*v*_ = 1.75 deg/sec and noise in the static feature system had a mean value of *μ*_*l*_ = 0 deg and an STD of *σ*_*l*_ = 1 deg. This resulted in the matched SNR values of 79.9 dB for the moving feature system and 80.1 dB for the static feature system. Because the mean value was zero, we call this the bias-free noise parameterization. In the second parameterization, noise in the moving feature system had a mean value of *μ*_*v*_ = 1 deg/sec and an STD of *σ*_*v*_ = 1.75 deg/sec and noise in the static feature system had the mean value of *μ*_*l*_ = 0.8 deg and an STD of *σ*_*l*_ = 1 deg. The matched SNR values are 75.7 dB and 75.6 dB for the moving feature system and static feature system, respectively. Because the mean value was nonzero, we call this the biased noise parameterization.

For the moving feature system, we estimate the linear velocity *v*_*z*_ and the rotational velocity *ω*_*y*_ (see Methods). In our model, these velocities transformed into Cartesian coordinates (using Eq 9) before integration of position. However, if we instead directly temporally integrate these velocities, noise in these two properties is characterized through a Brownian motion model, as illustrated in Fig 4A–4D. To derive the overall estimated radial distance *l* and the overall estimated angle *φ*, we multiply these angles by the temporal interval for a time step Δt: $l(t_{n})=\Delta t\sum_{i=1}^{n}v_{z,i}$ and $\varphi (t_{n})=\Delta t\sum_{i=1}^{n}\omega_{y,i}$, where *t*_*n*_ is the n-th time step and *i* indexes time steps. The ground-truth values are denoted by *l* for radial distance and *φ* for angle and the estimated values are denoted by $\hat{l}$ and $\hat{\varphi }$. We associate random variables *L*_*n*_ and *Φ*_*n*_ with the differences $l_{n}-{\hat{l}}_{n}$ and $\varphi_{n}-{\hat{\varphi }}_{n}$ for the time point *t*_*n*_. The variables *L*_*n*_ and *Φ*_*n*_ form a Brownian motion model, if these differences $l_{n}-{\hat{l}}_{n}$ and $\varphi_{n}-{\hat{\varphi }}_{n}$ are normally distributed. In our case, these distances are normally distributed and we associate the following normal distributions with $l_{n}-{\hat{l}}_{n}\in N(\mu_{l},\sigma_{l})$ and with $\varphi_{n}-{\hat{\varphi }}_{n}\in N(\mu_{\varphi },\sigma_{\varphi })$. Then, we get $(n-m)Var[L_{n}-L_{m}]=\frac{t_{n}-t_{m}}{\Delta t}\sigma_{l}^{2}+{\left(\frac{t_{n}-t_{m}}{\Delta t}\right)}^{2}\mu_{l}^{2}$, where Var is the statistical variance for these random variables *L*_*n*_ and *L*_*m*_. The same applies for the random variables *Φ*_*n*_ and *Φ*_*m*_. For our simulation, we used 100 trials to compare the Brownian motion model to the empirical measurements of the quantities of error in estimated distance and error in estimated angle. We measured *μ*_*l*,*noBias*_ = −4.07 × 10^−5^ *cm* and *σ*_*l*,*noBias*_ = 4.49 × 10^−2^ *cm*, for the estimate of distance based on linear velocity estimates and bias-free noise. The Brownian motion model fits well with the empirical measurements of radial distance error (Fig 4A). For the estimate of angle based on rotational velocity and bias-free noise, we measured *μ*_*φ*,*noBias*_ = −2.06 × 10^−5^ deg and *σ*_*φ*,*noBias*_ = 3.59 × 10^−2^ deg. The Brownian motion model and empirical measurements of angle error match (Fig 4B). For the biased noise, we get similar estimates for the radial distance error based on integration of linear velocity. These are *μ*_*l*,*Bias*_ = −8.09 × 10^−6^ *cm* and *σ*_*l*,*Bias*_ = 4.52 × 10^−2^ *cm*. The Brownian motion model and estimates match (Fig 4C). For the biased noise, we measure a bias in the estimates of angle based on rotational velocity as well, we get *μ*_*φ*,*Bias*_ = 4.99 × 10^−2^ deg and *σ*_*φ*,*Bias*_ = 3.60 × 10^−2^ deg. In this case, the squared error has the shape of a parabola, which originates from the 2^nd^ term in $(n-m)Var[\Phi_{n}-\Phi_{m}]=\frac{t_{n}-t_{m}}{\Delta t}\sigma_{\varphi }^{2}+{\left(\frac{t_{n}-t_{m}}{\Delta t}\right)}^{2}\mu_{\varphi }^{2}$ (Fig 4D).

In summary, the specific examples shown in Fig 4A–4D demonstrate that in the case of directly integrating linear velocity and rotational velocity, the accumulated error follows a Brownian motion model. However, in the model of position estimation used elsewhere in this paper (other than Fig 4A–4D), the linear and rotational velocities in polar coordinates are transformed into Cartesian coordinates to compute a position estimate (see first line of Eq 9). While noise in the radial distance estimates from linear velocity and angle estimates from rotational velocity adhere to a Brownian motion model, the integrated position estimate in Cartesian coordinates does not because of the coordinate transform from polar coordinates into Cartesian coordinates. Thus, the Euclidean distance error between the temporally integrated estimates based on velocities and ground-truth location does not increase proportional to √t (Fig 4E and 4I). Instead, the error reaches a plateau of 100 cm Euclidean distance after 10 seconds. The static feature system, similarly reaches errors of up to 100 cm within the first 10 secs (Fig 4F and 4J). For the biased noise parameterization the moving feature system accumulates errors up to ≈ 200 cm, which is close to the maximum possible error of √2 × 150 cm or 212.13 cm (Fig 4G and 4K). The error for the static feature system in the biased noise parameterization is very similar to that of the bias-free noise parameterization (Fig 4H and 4L).

Next, we wanted to study the influence of these errors on the grid cell firing patterns. For this simulation, we used the velocity controlled oscillator model supplied with temporally integrated, estimated velocities or directly with location estimates. We simulated grid cells for 2,500 seconds or 41.7 minutes. Results of grid cell responses using the attractor model [18] are qualitatively the same (S2 Fig). In the attractor models, we provided input consisting of the temporal difference of the sequential locations as a velocity signal input. Notice that in general the broad category of attractor models can work with either position or velocity input, but the particular model [18] that we used requires velocities as input. For the bias-free noise the grid patterns are intact for both the moving feature system (Fig 4M) and for the static feature system (Fig 4N). For the biased noise, the grid pattern for the moving feature system disappears (Fig 4O), and the firing pattern would not be considered that of a grid cell. The grid pattern for the static feature system remains intact (Fig 4P). Thus, the grid cells driven by the static feature system can tolerate more biased noise measured through the SNR than grid cells driven by the moving feature system. We ran simulations to determine the limits of noise tolerance, where we ceased to observe recognizable grid cell spatial firing fields. For the bias-free noise the limits of noise tolerance are *σ*_*v*_ ≈ 5 deg/sec for the moving feature system and *σ*_*l*_ ≈ 22 deg for the static feature system. For biased noise these limits are *μ*_*v*_ ≈ 0.01 deg/sec and *σ*_*l*_ ≈ 1.75 deg/sec for the moving feature system and *μ*_*l*_ ≈ 15 deg and *σ*_*l*_ ≈ 18 deg for the static feature system. These noise limits illustrate the major difference between the moving and static feature system: The moving feature system integrates error over time while the static feature system does not integrate error. The integration of error is especially detrimental to the grid cell firing using estimates of the moving feature system in the case of biased noise (Fig 4O).

## Discussion

Here, we demonstrate how different grid cell modules in entorhinal cortex might be influenced by different mechanisms for the estimation of a rat’s location in an environment based on visual features. The first mechanism uses optic flow (moving feature system) to estimate and integrate self-motion velocities to determine the rat’s allocentric location in an environment. The second mechanism uses visual landmarks (static feature system) to triangulate the rat’s allocentric location in an environment. We use these location estimates as input to a model of grid cell firing to generate firing fields within the environment. In our model, we associate the processing of location based on moving visual features (optic flow) with input to grid cells in dorsal, medial entorhinal cortex—possibly due to stronger input from the dorsal retinotopic areas that code the ventral visual field that contains the ground plane. We associate the processing of location based on static visual features (landmarks) with input to grid cells in more ventral, medial entorhinal cortex (possibly due to stronger input from ventral retinotopic areas that code the dorsal visual field processing wall features). This model with its two mechanisms simulates the absence of compression of the grid cell firing fields recorded in cells of dorsal modules with narrow spacing between the firing fields because the optic flow information does not depend on the compression of barriers. The model also simulates the compression of the grid cell firing fields recorded in more ventral modules with wider spacing between the firing fields because static visual features (landmarks) on the compressed barriers change the sensed location of the rat in the environment. Thus, our model provides an explanation for differences in compression properties of grid cells recorded from different modules in medial entorhinal cortex [9].

In our model, we presented the dorsal and ventral module as the two boundary points of a spectrum of possible modules. In the data [9] there are intermediate modules that are influenced by wall compression to different extents. Our model could generalize to such intermediate modules using a linear superposition between the location estimates of the moving feature system and the static feature system.

We also studied how noise in visual angle would alter the location coding by the moving feature system and the static feature system. For the moving feature system, we superimposed noise onto the observed velocities in azimuth and elevation angle (Eq 19). This noise leads to a normally distributed error in the estimated linear velocity and rotational velocity. And as this error is integrated in the estimates of radial distance and angle from the linear and rotational velocity, the error in these two dimensions is characterized through a Brownian motion model. Thus, the square distance of the error in estimates is proportional to the time of integration (Fig 4A–4D). However, when these properties of linear and rotational velocity are transformed from polar coordinates into Cartesian coordinates to define a position estimate (Eq 9), then the build-up over time of position error in Cartesian coordinates is not any more characterized by a Brownian motion model (Fig 4E–4H). For the static feature system, we superimposed noise onto the observed visual angles in azimuth and elevation (Eq 20). Our triangulation method directly estimates the position from these azimuth and elevation angles and no temporal integration occurs for the estimated position. Thus, error does not carry over from one time step to the next and this system is not characterized by a Brownian motion model.

The difference in compression between the moving feature system and the static feature system in these simulations depends entirely on the mechanism computing location using visual features. The chosen grid cell models do not contribute to the presence or to the absence of compression. We added simulations of grid cell models using the location estimates from our model to further visualize the compression on grid cell models and to allow for a direct comparison to compression effects recorded from grid cells.

The simulation results for grid cell models (Fig 3) do not depend on the specific grid cell firing model used. To demonstrate this, we replicated the results from Fig 3 by using the attractor model of grid cells [18] instead of the velocity controlled oscillator [6] (S1 Fig). Thus, this paper is neutral with regard to the use of grid cell models based on oscillatory interference [6, 16, 17] versus models based on attractor dynamics [5, 19] or models using both properties [20].

Here, we do not focus on a specific neurobiological mechanism for the influence of location or velocity estimates on grid cell firing. In attractor models, the influence of location or velocity is attributed to the influence of conjunctive grid-by-head-direction cells [5, 18, 19]. In velocity controlled oscillator models, the influence of location or velocity is attributed to the influence on oscillator frequency, which alters phase over time. Reset of oscillatory phase based on putative place cell input has been used to model experimental data on compression of grid cell firing fields [8] in a hybrid model of attractor dynamics and oscillatory interference [20], but that hybrid model did not directly model the role of visual features or the differential effect on different modules.

Formally, publications of the velocity controlled oscillator model [6, 16, 17] include the integration term for the velocity projected onto a basis system of three vectors in a plane, e.g. at 120 degrees angular difference. We separated out this temporal integration (Eqs 16 and 9). For the dorsal grid cells in our model the optic flow signal could either be used as a velocity input or temporally integrated to provide a location input. Our triangulation method provides a location signal (Eq 14) and we assume that the role of grid cells is to form a distributed, error-correcting, representation using this location signal.

Constraints in the environment may favor different methods for the estimation of location on different spatial scales, meaning different spatial distances to features in the box. For instance, during visual navigation, visual features that are close to the animal produce reliable optic flow, because these close features produce large retinal flow vectors, which are less sensitive to noise. It follows that the estimation of translational self-motion is most reliable with sample points near to the animal. In an open field environment, used in studies of grid cells during rat foraging [1, 2, 8, 9], such near sample points are located on the ground plane close to the foraging rat. Our model follows this idea and uses optic flow of moving visual features from the ground plane for the estimation of self-motion. In contrast, the estimation of location through triangulation is more reliable for distant, static visual features (landmarks) as the perturbation of distant static features in their location has only a small effect on the visual angle at which these appear. In our simulations, our static feature system uses landmarks on barriers for triangulation, and these landmarks appear mostly as distant from the rat.

This description raises the interesting possibility that the difference in coding within dorsal versus ventral medial entorhinal cortex corresponds to coding of different components of the visual field. The visual cortical areas on the dorsal surface of the rodent brain show retinotopic mapping [10, 14], and it is possible that a similar retinotopic mapping may map the dorsal portions of the retina to the dorsal portions of the medial entorhinal cortex. Due to the inversion of visual images in the eye, the dorsal retina would respond more to the ventral visual field or ground plane near the animal. Thus, the dorsal portions of medial entorhinal cortex may respond to feature locations on the ground near the animal, which are less likely to be affected by barrier location. Similarly, in this framework, the ventral portions of the retina might be mapped to more ventral portions of the medial entorhinal cortex. The ventral retina would respond to more dorsal portions of the visual field that would include elevated features on barriers. Thus, the ventral portions of medial entorhinal cortex might respond to feature locations on the distant barriers, and therefore be more sensitive to the location of such barriers. These effects of retinotopic input to medial entorhinal cortex could underlie the differential coding of a moving feature system for dorsal regions and static feature system for ventral regions. If this is the case, it could also be interpreted that static visual features are used for location estimation in all portions of the visual field. The difference in compression seen for grid cells in different modules might arise because visual features on the ground are not altered by the movement of the barriers. This could be tested by adding visual features on the ground (markings) and experimentally compressing the location of these visual features on the ground to determine if this could cause compression in the dorsal grid cells appearing as narrower spacing between their firing fields.

Consistent with the above proposal, recent data from rodents shows that responses of different visual regions are restricted to specific portions of the visual field [14]. Area RL in the rodent responds to the ventral visual field (ground plane), whereas area LM responds to the dorsal visual field (where distal barriers would appear). In addition, anatomical data shows selectivity in the connections from these visual regions to the dorsal entorhinal cortex [12]. In that paper, one figure showing the spread of anterograde tracers from areas AL/RL indicates projections to dorsal MEC, while another figure shows that area LM does not project to dorsal MEC [12].

During the course of the development of these simulations, a number of different mechanisms were tested before it was found that the best match to experimental data [9] was obtained with the specific mechanism using a compression factor for the location computation using opposite walls. Alternate mechanisms, like triangulation without regularization or triangulation using individual pairs of static visual features from the same barrier did not match the compression effects seen in the data. Though smaller scales of compression could be obtained, these alternate mechanisms could not achieve the 90% compression necessary to match the data [9]. To get large scale compression, it was necessary to use the framework of a compression factor dependent upon individual static features mapped on opposite barriers. This representation of features on barriers could be partly due to the maintenance of a boundary cell response even when an animal turns away from the barrier—so it could look at one barrier and then maintain that boundary cell response when viewing a different barrier. This capacity to maintain a working memory of static visual features could depend upon intrinsic mechanisms for persistent spiking observed in neurons of medial entorhinal cortex [21, 22, 23].

The rodent visual system also has regions that respond differentially to the nature of visual input, with some regions responding more strongly to high temporal frequency (moving stimuli) with low spatial frequency (e.g. area AM), and others responding more to low temporal frequency (more static stimuli) with higher spatial frequency (e.g. area PM), and along this continuum other areas show differences in response to temporal and spatial frequency (e.g. AL, RL, LM) [11, 12, 13, 24, 25]. These responses might indicate differential processing in different rodent visual systems in a manner similar to the distinction between the “where” and “what” pathways described extensively for the dorsal and ventral streams of the primate cortex [15]. Note that the primate extrastriate visual cortex contains regions that explicitly respond to the self-motion indicated by optic flow. Thus, visual areas in rodent might have some features analogous to the primate regions [26–28]. In particular, neurons in primates were shown to be responsive to the pattern flow caused by two components [29], and a small number of neurons in area AL and RL of rodents show similar motion selectivity [30]. Our simulations suggest that this distinction might correspond to differential mechanisms of processing in the dorsal medial entorhinal cortex versus the ventral medial entorhinal cortex. Our model links the processing of optic flow to the dorsal medial entorhinal cortex and the processing of landmarks to the ventral medial entorhinal cortex. Notice that the distinction between the dorsal and ventral streams in the primate cortex involves many more functional regions and has not been applied to entorhinal cortex.

Previous discussions of “where” vs. “what” pathways in the entorhinal cortex have focused on the notion that the “where” pathway would involve input from postrhinal cortex to medial entorhinal cortex and the “what” pathway would involve input from perirhinal cortex to lateral entorhinal cortex [31, 32]. Consistent with this, experimental data, selectivity for objects appears in lateral entorhinal cortex [33]. Previous studies showed stronger visual input to medial entorhinal cortex compared to lateral entorhinal cortex in the rat [34], but surprisingly recent analysis of network anatomical interactions in mice suggest equal ventral stream “what” input to lateral and medial entorhinal cortex with stronger “where” input to lateral entorhinal cortex [25].

The distinction between lateral, representing the “what”, and medial entorhinal cortex, representing the “where”, is a hypothesis that has not yet been fully tested. However, we would like to note that the evaluation of differences between lateral and medial entorhinal cortex is a separate issue from our proposal of differences between coding in dorsal versus ventral portions of medial entorhinal cortex. Lateral entorhinal cortex is not addressed in our simulations. Our simulations instead focus on the potential interest in analyzing differences in the inputs to the dorsal and ventral portions of medial entorhinal cortex itself, as these may differ. These inputs may differ in the amount of input from different visual regions coding moving versus static visual features [11–14, 24, 30]. As an additional possibility, anatomical data consistently shows that the visual regions in the rodent show selective retinotopic mapping within each visual region [10, 12–14]. Our simulations may indicate that this differential mapping of different portions of the visual field may extend to influence the nature of processing in different portions of the medial entorhinal cortex.

## Methods

Our model has two functionally different streams, which consist of a moving feature system and a static feature system (see Fig 1). As input to these systems, we simulated the visual input that would be viewed by a rat on a synthesized, random trajectory. This visual input used a simplified ray-tracer and a spherical camera model. In the moving feature system, visual motion is processed in the form of optic flow. This optic flow information is used to estimate the linear and rotational velocity of the rat. An integration of these velocity estimates results in a location estimate. In the static feature system of our model, visual landmarks are processed. We used these landmarks to estimate the location of self through triangulation. The estimated location information from both streams is forwarded to a model for grid cell firing in the entorhinal cortex. For this example, we used the oscillatory interference model for simplicity, but similar effects could be obtained with most other types of grid cell models such as an attractor model of grid cells (S1 Fig). We summarized all model parameters in Table 1. A Matlab implementation of the model including the capability to simulate the visual input is provided through S1 File.

**Table 1:** Default parameter values for the synthesized trajectories, spherical camera model, and module model. Some simulations have changed parameter values and we denoted this explicitly.

Description of parameter	Identifier and value
Synthesized trajectories inside a cube	
Number of samples	*n*_*Sample*_ = 50,000
Sampling frequency	*f*_*Sample*_ = 20 Hz
Duration of the simulated time	*T* = 41.67 min
Peak location *β*_*v*_ of the Rayleigh distribution	*β*_*v*_ ≈ 13 cm/sec
Mean of the normal distribution	*μ*_*ω*_ ≈ 0 deg/sec
Standard deviation of the normal distribution	*σ*_*ω*_ ≈ 340 deg/sec
Width and length of the square environment	*w*_*Cube*_ = *l*_*Cube*_ = 150 cm
Height of walls	*h*_*Cube*_ = 50 cm
Eye-height above ground	*d* = 2.5 cm
Number of visual features on ground, walls, and ceiling	*n*_*Features*_ = 54
Number of samples on the ground	*n*_*Ground*_ = 9
Spherical camera model	
Horizontal field of view	*h*_*FoV*_ = 360 deg
Vertical field of view	*v*_*FoV*_ = 120 deg
Focal length	*f*_*Sphere*_ = 1 cm
Module model	
Initial head direction	*φ*_*0*_ = 0 deg
Initial location	(*x*_*0*_,* y*_*0*_) = (0 cm, 0 cm)
Regularization parameter	*α* = 10^−4^
Velocity controlled oscillator model	
Oscillation of theta rhythm	*f* = 7.38 Hz
Parameter for grid spacing–moving feature system	*β*_*1*_ = 0.004
Parameter for grid spacing–static feature system	*β*_*2*_ = 0.003
Threshold for spikes	*ϴ* = 1.8
Attractor model	
Horizontal cells	*n*_*x*_ = 9
Vertical cells	*n*_*y*_ = 10
Synaptic peak strength	*I* = 0.3
Inhibition for weights	*T* = 0.05
Input gain (grid spacing)–moving feature system	*α*_*1*_ = 1.4×10^−3^
Input gain (grid spacing)–static feature system	*α*_*2*_ = 0.9×10^−3^
Gaussian standard deviation	*σ* = 0.24 meters
Grid orientation	*γ* = 0°
Threshold for firing	*η* = 0.1
Simulations	
Standard deviation of velocity noise	*σ*_*v*_ in units of deg/sec
Standard deviation of location noise	*σ*_*l*_ in units of deg/sec
Mean of velocity noise	*μ*_*v*_ in units of deg/sec
Mean of location noise	*μ*_*l*_ in units of deg/sec

### Synthesized rat trajectories inside a box

We simulated the rat movement within a box that has two configurations. In configuration A the box has the dimensions 150 cm × 150 cm and in configuration B the box has been altered by moving barriers to have the dimensions 150 cm × 100 cm. Barriers are 50 cm high. On the ground, ceiling, and the four barriers or side-walls, we randomly distributed 9 features per surface. In total, there are *n*_*Features*_ = 9 × 6 = 54 features. Due to the limited field of view of the rat of 120 deg vertically, not all these features are visible at all times. To guarantee that features from both barrier pairs are available to the rat, we assumed that the angles of these features are available as input for 360 deg, horizontally. This can be achieved, e.g., through updating of short term memory for feature angle based on subsequent turns of the head. For example, a feature viewed at 90 degrees could be maintained in short term memory and updated by a subsequent 90 degree head turn, so that the animal still has knowledge about the features at 180 degrees.

We synthesized rat trajectories based on the movement statistics of recorded rat trajectories [35, 36, 37]. We modeled the movement statistics by fitting forward linear speeds (along the optical axis) through a Rayleigh distribution, which gave the peak location *β*_*v*_ = 13.25 cm/sec. In addition, we fitted rotational yaw-speeds by a normal distribution, which gave the mean *μ*_*ω*_ = 0°/sec and standard deviation (STD) *σ*_*ω*_ = 337.93°/sec [38; their Fig 2]. We used these distributions to draw samples for linear and rotational speeds, respectively, for all 50,000 sample points. When no collision with a barrier occurs, translational and rotational speeds were selected from generated data. A collision occurs if the simulated rat is within 15 cm distance of the closest barrier and the angular difference between the direction of travel and the normal direction of the “facing” planar barrier is smaller than 90 deg. Then, we use the following two-step maneuver for collision avoidance. First, we reduced the speed by half of the difference between the current speed and the minimal speed, which is 5 cm/sec. Second, we rotated away from the barrier that poses a collision threat. The rotation angle is the angle between the direction of travel and the normal direction of the planar barrier that poses a collision threat. To avoid periodic turning in corners, we added a randomized rotation value to the rotation angle. For the synthesized trajectory of the box in configuration A we measured *β*_*v*_^*m*^ = 13.17 cm/sec and *σ*_*ω*_^*m*^ = 342.49 deg/sec and in configuration B we measured *β*_*v*_^*m*^ = 13.14 cm/sec and *σ*_*ω*_^*m*^ = 357.07 deg/sec. All fitted values are close to the values of recorded rat trajectories, which are *β*_*v*_ = 13.25 cm/sec and *σ*_*ω*_ = 337.93°/sec.

### Estimation of self-motion from image velocities

In these simulations, there were two methods of estimating location, proposed to represent differences in neural properties of the different modules in entorhinal cortex. These two methods consist of either estimating location from image feature velocities (optic flow) in our moving feature system or from triangulation of image features in our static feature system. The exact biological mechanism of these differences between modules are not explicitly modeled, but they could arise from the nature of the regions providing input to different modules, or possibly from other factors such as the location of features in the visual field or the spatial distance of the input features.

In this section, we describe the first method (the moving feature system), which estimated location based on self-motion from image velocities, that is, from the movement of visual features. All features on the surfaces of the box are described by their 3D point locations. The 3D point locations ${\vec{P}}_{i}=(X_{i},Y_{i},Z_{i})$ are described by the azimuth *θ*_*j*_ and elevation *ϕ*_*j*_ coordinates using

$$\left(\begin{array}{c} \theta_{i}\\ \phi_{i} \end{array}\right)=\left(\begin{array}{c} \text{arctan}\;2(X_{i},Z_{i})\\ \text{arctan}(Y_{i}/\sqrt{X_{i}^{2}+Z_{i}^{2}}) \end{array}\right)\;\text{for}\;i=1\ldots n_{Visible}.$$ (1)

These angles *θ*_*j*_ and *ϕ*_*j*_ are invariant to the distance of the sample point. Each of these visible features introduces an image velocity defined by the angular velocity for the azimuth angle ${\dot{\theta }}_{i}$ and the angular velocity for the elevation angle ${\dot{\phi }}_{i}$, assuming that the rat moves with the linear velocity $\vec{v}=(v_{x},v_{y},v_{z})$ and rotational velocity $\vec{\omega }=(\omega_{x},\omega_{y},\omega_{z})$. To derive the model for the image velocity, we assumed a spherical camera model. This spherical camera model with the radius *f*_*Sphere*_, projects 3D point locations ${\vec{P}}_{i}=(X_{i},Y_{i},Z_{i})$ onto the spherical surface locations ${\vec{p}}_{i}=(x_{i},y_{i},z_{i})$ using the model equation:

$$\left(\begin{array}{c} x_{i}\\ y_{i}\\ z_{i} \end{array}\right)=\frac{f_{Sphere}}{D_{i}}\left(\begin{array}{c} X_{i}\\ Y_{i}\\ Z_{i} \end{array}\right)\;\text{with}\;D_{i}=\sqrt{X_{i}^{2}+Y_{i}^{2}+Z_{i}^{2}}.$$ (2)

Assuming instantaneous motion for the self-motion, the movement of the sampled point locations ${\vec{P}}_{i}$ is modeled by ${\dot{\vec{P}}}_{i}=\vec{v}-{\vec{P}}_{i}\times \vec{\omega }$ [39]. In addition, we use the spherical coordinates

$$\left(\begin{array}{c} x_{i}\\ y_{i}\\ z_{i} \end{array}\right)=f_{Sphere}\;\left(\begin{array}{c} \text{sin}\;\theta_{i}\;\text{cos}\;\phi_{i}\\ \text{sin}\;\phi_{i}\\ \text{cos}\;\theta_{i}\;\text{cos}\;\phi_{i} \end{array}\right),$$ (3)

with the z-axis pointing along the direction of travel. Note, that this z-axis is typical in computer vision research, but it contrasts with the usual direction of the z-axis in describing rat behavioral data during grid cell firing, in which the z-axis is the direction orthogonal to the ground-plane.

Calculating the temporal differential for Eq 2 allows us to plug in the instantaneous motion model ${\dot{\vec{P}}}_{i}$. Then, we use Eq 3 and calculate the temporal differentials to link the result to the angular velocities. The two resulting equations are relate to each other through the common expression ${\dot{\vec{p}}}_{i}$. Solving for the angular velocities, we get the model equation [38]

$$\begin{array}{c} \left(\begin{array}{c} {\dot{\theta }}_{i}\\ {\dot{\phi }}_{i} \end{array}\right)=\frac{1}{D}\left(\begin{array}{ccc} -\frac{\text{cos}\;\theta_{i}}{\text{cos}\;\phi_{i}} & 0 & \frac{\text{sin}\;\theta_{i}}{\text{cos}\;\phi_{i}}\\ \text{sin}\;\theta_{i}\;\text{sin}\;\phi_{i} & -\text{cos}\;\phi_{i} & \text{cos}\;\theta_{i}\;\text{sin}\;\phi_{i} \end{array}\right)\left(\begin{array}{c} v_{x}\\ v_{y}\\ v_{z} \end{array}\right)\\ +\left(\begin{array}{ccc} \frac{\text{sin}\;\theta_{i}\;\text{sin}\;\phi_{i}}{\text{cos}\;\phi_{i}} & -1 & \frac{\text{cos}\;\theta_{i}\;\text{sin}\;\phi_{i}}{\text{cos}\;\phi_{i}}\\ \text{cos}\;\theta_{i} & 0 & -\text{sin}\;\theta_{i} \end{array}\right)\left(\begin{array}{c} \omega_{x}\\ \omega_{y}\\ \omega_{z} \end{array}\right) \end{array}.$$ (4)

As simplifications, we assume that the rat looks straight forward, tangent to the running direction, thus, *v*_*x*_ = 0 cm/sec and *v*_*y*_ = 0 cm/sec, and we assume that the rat undergoes neither pitch nor roll rotations, thus, *ω*_*x*_ = 0 deg/sec and *ω*_*y*_ = 0 deg/sec.

In addition to these properties, we assume that all computation of image velocities in the moving feature system originate from features on the ground plane, which we propose may be the primary input for certain modules in entorhinal cortex. These features can be described through the plane equation (*n*_*x*_, *n*_*y*_, *n*_*z*_)(*X*_*i*_, *Y*_*i*_, *Z*_*i*_)^*T*^ − *d* = 0 with the normal vector (*n*_*x*_, *n*_*y*_, *n*_*z*_) the point location (*X*_*i*_, *Y*_*i*_, *Z*_*i*_) and the distance *d* from the origin measured along the normal direction. We use the uppercase *T* in the super-script as transpose operator. Using Eq 2 and Eq 3 we find the following expression for the distance

$$D_{i}=\frac{d}{n_{x}\;\text{sin}\;\theta_{i}\;\text{cos}\;\phi_{i}+n_{y}\;\text{sin}\;\phi_{i}+n_{z}\;\text{cos}\;\theta_{i}\;\text{cos}\;\phi_{i}}.$$ (5)

Plugging in the simplifications, we got the model for spherical image velocities given curvilinear motion above a ground plane as

$$\left(\begin{array}{c} {\dot{\theta }}_{i}\\ {\dot{\phi }}_{i} \end{array}\right)=\frac{\text{sin}\;\phi_{i}}{d\;\text{cos}\;\phi_{i}}\left(\begin{array}{c} \text{sin}\;\theta_{i}\\ \text{cos}\;\theta_{i}\;\text{sin}\;\phi_{i}\;\text{cos}\;\phi_{i} \end{array}\right)v_{z}+\left(\begin{array}{c} -\omega_{y}\\ 0 \end{array}\right).$$ (6)

We assume that the sensed image velocity or optic flow is defined by the variables ${\dot{\hat{\theta }}}_{i}$ for the azimuth angle and ${\dot{\hat{\phi }}}_{i}$ for the elevation angle and is given. In addition, the height *d* of the rat’s eyes off the ground is also given. Then, we want to estimate the unknown linear velocity *v*_*z*_ and rotational velocity *ω*_*y*_. We use a least squares approach to define the optimization problem

$${\text{arg min}}_{v_{z},\omega_{y}}\underset{=:F(v_{z},\omega_{y})}{\underset{︸}{\sum_{i=1}^{n_{Ground}}{\left\|\left(\begin{array}{c} {\dot{\hat{\theta }}}_{i}\\ {\dot{\hat{\phi }}}_{i} \end{array}\right)-\frac{\text{sin}\;\phi_{i}}{d\;\text{cos}\;\phi_{i}}\left(\begin{array}{c} \text{sin}\;\theta_{i}\\ \text{cos}\;\theta_{i}\;\text{sin}\;\phi_{i}\;\text{cos}\;\phi_{i} \end{array}\right)v_{z}+\left(\begin{array}{c} -\omega_{y}\\ 0 \end{array}\right)\right\|}^{2}}}$$ (7)

with the functional *F*. We calculate the extremal value for this functional *F*, which are given by the solution to the linear equation system

$$\left(\begin{array}{cc} a_{11} & a_{12}\\ a_{21} & a_{22} \end{array}\right)\left(\begin{array}{c} {\hat{v}}_{z}\\ {\hat{\omega }}_{y} \end{array}\right)=\left(\begin{array}{c} b_{1}\\ b_{2} \end{array}\right)\;\text{with}$$ (8)

$$a_{11}=\frac{1}{d}\frac{1}{n_{Ground}}\sum_{i=1}^{n_{Ground}}{\text{tan}}^{2}\;\phi_{i}\;{\text{sin}}^{2}\;\theta_{i}+{\text{sin}}^{4}\;\phi_{i}\;{\text{cos}}^{2}\;\theta_{i}\;,$$

$$a_{12}=-\frac{1}{n_{Ground}}\sum_{i=1}^{n_{Ground}}\text{tan}\;\phi_{i}\;\text{sin}\;\theta_{i},\;a_{21}=-a_{12},\;a_{22}=-1,$$

$$b_{1}=\frac{1}{n_{Ground}}\sum_{i=1}^{n_{Ground}}{\dot{\hat{\theta }}}_{i}\;\text{tan}\;\phi_{i}\;\text{sin}\;\theta_{i}+{\dot{\hat{\phi }}}_{i}\;{\text{sin}}^{2}\;\phi_{i}\;\text{cos}\;\theta_{i},\;b_{2}=\frac{1}{n_{Ground}}\sum_{i=1}^{n_{Ground}}{\dot{\hat{\theta }}}_{i}\;,$$

where *n*_*Ground*_ refers to the number of all visible features on the ground and the index *i* ranges over all these features. The inverse of matrix A is defined for non-degenerated cases, e.g. *ϕ*_*i*_ = 0. We use the solution of Eq 8 to estimate the linear velocity and rotational yaw-velocity.

For a location estimate, we temporally integrated estimates of the linear and rotational velocity. For a time discrete sampling, we defined the estimated linear velocities by ${\hat{v}}_{z,j}$ and the estimated rotational velocities by ${\hat{\omega }}_{y,j}$ where *j* indexes the sampled time in steps of ∆t = 0.05 sec or *f*_*Sample*_ = 20 Hz. We calculated the estimated location in the ground-plane through the x-coordinate and y-coordinates by

$${\hat{x}}_{m}=\sum_{j=1}^{m}\Delta t\;{\hat{v}}_{z,j}\;\text{cos}\;\varphi_{j}+x_{0}\;\text{and}\;{\hat{y}}_{m}=\sum_{j=1}^{m}\Delta t\;{\hat{v}}_{z,j}\;\text{sin}\;\varphi_{j}+y_{0}\;\text{for}$$ (9)

$$\varphi_{n}=\sum_{j=1}^{n}\Delta t\;{\hat{\omega }}_{z,j}+\varphi_{0}.$$

The values *x*_*0*_, *y*_*0*_, and *φ*_*0*_ denote the initial location of the rat and its initial head direction, respectively.

### Estimation of location through regularized triangulation

**Fig 5:**
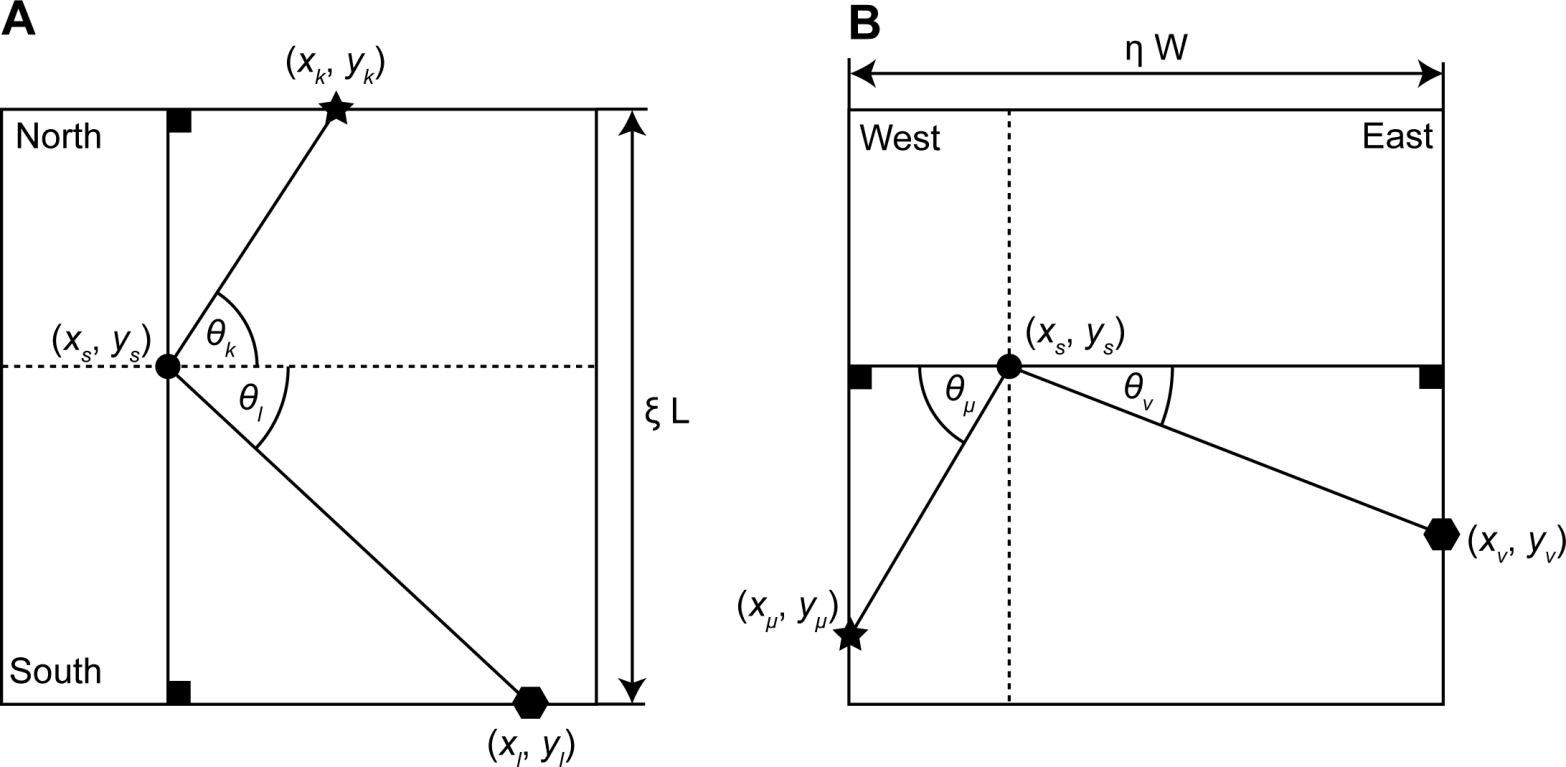
Shows the geometric constraints of the model for the static feature system. (A) Constraint for features sampled on northern or southern wall. (B) Constraint for features sampled on western or eastern wall.

The second method of location estimation (the static feature system) uses a regularized triangulation based on visual features. This method can model the proportional compression of the estimated location in the ground plane according to the shift of features on the side walls. For regularization, we used the constraint of a fixed, known width *W* and fixed, known length *L* of the box. We used trigonometric constraints to express this length and width of the box using all pair-wise sample points from opponent walls (Fig 5). The unknown intersection point is defined by (*x*_*s*_, *y*_*s*_) and the memorized locations are defined by (*x*_*k*_, *y*_*k*_), (*x*_*l*_, *y*_*l*_), (*x*_*μ*_, *y*_*μ*_), or (*x*_*ν*_, *y*_*ν*_) for the northern, southern, western, or eastern wall, respectively. We used four indices to refer to the sampled features on the four walls. We indexed sampled features on the northern wall by *k*, features on the southern wall by *l*, features on the western wall by *μ*, and features on the eastern wall by *ν*. We used the sub-script *k˄l* to index all features from the northern and southern wall and we used the sub-script *μ˄ν* to index all features from the western and eastern wall. In contrast, we indexed all pairs of sampled features from the northern and southern wall by the sub-script *k*,*l* and for all pairs of sampled features on the western and eastern wall by the sub-script *μ*,*ν*.

To match the observed, azimuth angles for the sample points defined in Eq 1 with the defined angles *θ*_*k*_, *θ*_*l*_, *θ*_*µ*_, and *θ*_*ν*_ (compare with Fig 5) we have to use the following transformations *θ*_*k*_ = *θ*_*i*_ ∧ *i* ∈ *S*_*N*_, *θ*_*l*_ = −*θ*_*i*_ ∧ *i* ∈ *S*_*S*_, *θμ* = *θ*_*i*_ – *π*/2 ∧ *i* ∈ *S*_*W*_, and *θ*_*ν*_ = −*θ*_*i*_ – *π*/2 ∧ *i* ∈ *S*_*E*_. The identifiers *S*_*N*_, *S*_*S*_, *S*_*W*_, and *S*_*E*_ denote sets of indices for sampled points on the northern, southern, western, and eastern wall, respectively.

We introduce the two compression variables *η* and *ξ* for the compression in the x-dimension and the y-dimension, respectively (Fig 5). Taking an arbitrary point pair from the northern and southern wall, we get the following constraint

$$(x_{s}-x_{k,l})\;\text{tan}\;\theta_{k}+(x_{s}-x_{k,l})\;\text{tan}\;\theta_{k,l}=\xi \;L.$$ (10)

This constraint Eq 10 uses the two triangles that are defined through the corner locations (*x*_*s*_, *y*_*s*_) and the two feature locations (*x*_*k*_, *y*_*k*_) and (*x*_*l*_, *y*_*l*_) and their observed angles *θ*_*k*_ and *θ*_*k*_. The constraint for the western and eastern wall is analogously defined. Based on the observed angles, the length of the box may appear as shorter than the triangulation of the memorized locations suggest. This deflection between observed angles and memorized locations is modeled through the compression factor 0 < *ξ* ≤ 1. In all cases, we assume that the observed feature locations that are described by the angle *θ* have been re-referenced to an allocentric system using the initial head direction *φ*_*0*_.

The next constraint is the triangulation constraint. For this constraint, we denote the unknown length toward a sampled feature location by *λ*. When taking the compression variables into account, we get the following equations for each sampled feature:

$$x_{s}-x_{k\wedge l}-\lambda_{k\wedge l}\;\text{cos}\;\theta_{k\wedge l}=0\;\text{or}\;x_{s}-\eta \;x_{\mu \wedge \nu }-\lambda_{\mu \wedge \nu }\;\text{cos}\;\theta_{\mu \wedge \nu }=0\;\text{and}$$

$$y_{s}-\xi \;y_{k\wedge l}-\lambda_{k\wedge l}\;\text{sin}\;\theta_{k\wedge l}=0\;\text{or}\;y_{s}-y_{\mu \wedge \nu }-\lambda_{\mu \wedge \nu }\;\text{sin}\;\theta_{\mu \wedge \nu }=0.$$ (11)

Only two of these four equations apply for each sample point. If the sample point is on the western or eastern wall, then the first equation of each line applies; otherwise, the second equation of each line applies.

We combined all four constraints to define the functional

$$\begin{array}{l} E(x_{s},y_{s},\eta ,\xi ,\lambda_{k\wedge l},\lambda_{\mu \wedge \nu })\\ \;=\frac{1-\alpha }{n_{N}+n_{S}}\sum_{k\wedge l}^{}{\left(x_{s}-x_{k\wedge l}-\lambda_{k\wedge l}\;\text{cos}\;\theta_{k\wedge l}\right)}^{2}+{\left(y_{s}-\xi \;y_{k\wedge l}-\lambda_{k\wedge l}\;\text{sin}\;\theta_{k\wedge l}\right)}^{2}\\ \;+\frac{\alpha }{n_{N}n_{S}}\sum_{k,l}^{}{\left(\left(x_{s}-x_{k}\right)\text{tan}\;\theta_{k}+\left(x_{s}-x_{l}\right)\text{tan}\;\theta_{l}+\xi \;L\right)}^{2}\\ \;+\frac{1-\alpha }{n_{W}+n_{E}}\sum_{\mu \wedge \upsilon }^{}{\left(x_{s}-\eta \;x_{\mu \wedge \upsilon }-\lambda_{\mu \wedge \nu }\;\text{cos}\;\theta_{\mu \wedge \upsilon }\right)}^{2}+{\left(y_{s}-y_{\mu \wedge \upsilon }-\lambda_{\eta \wedge \upsilon }\;\text{sin}\;\theta_{\mu \wedge \nu }\right)}^{2}\\ \;+\frac{\alpha }{n_{W}n_{E}}\sum_{\mu ,\nu }^{}{\left(\left(y_{s}-y_{\mu }\right)\text{tan}\;\theta_{\mu }+\left(y_{s}-y_{\upsilon }\right)\text{tan}\;\theta_{\upsilon }+\eta \;W\right)}^{2} \end{array}.$$ (12)

As a regularization parameter we chose *α* to weigh the influence between the triangulation constraint by (1-*α*) and we weigh the influence of the constraint based on the dimensions of the box by *α*. The number of samples on the walls is given by *n*_*N*_, *n*_*S*_, *n*_*W*_, and *n*_*E*_ for the northern, southern, western, and eastern wall respectively. In Eq 12, the first and third term come from the triangulation constraint and the second and fourth term come from the regularization constraint on a fixed width and length of the box. In total, we have 4 + *n*_*Wall*_ unknowns, whereas *n*_*Wall*_ denotes all visible features on walls. We calculated the extreme value for the functional *E* by computing the partial derivatives of *E* for all unknowns. This results in 4 + 2*n*_*Wall*_ equations. The solution is calculated by using the following linear equation system

$$\left(\begin{array}{cccc} c_{11} & c_{12} & c_{13} & c_{14}\\ c_{21} & c_{22} & c_{23} & c_{24}\\ c_{31} & c_{32} & c_{33} & c_{34}\\ c_{41} & c_{42} & c_{43} & c_{44} \end{array}\right)\left(\begin{array}{c} {\hat{x}}_{s}\\ {\hat{y}}_{s}\\ \hat{\eta }\\ \hat{\xi } \end{array}\right)=\left(\begin{array}{c} d_{1}\\ d_{2}\\ d_{3}\\ d_{4} \end{array}\right)\;\text{with}$$ (13)

$$\begin{array}{l} c_{11}=\frac{1-\alpha }{n_{N}+n_{S}}\sum_{k\wedge l}1-{\text{cos}}^{2}\;\theta_{k\wedge l}+\frac{1-\alpha }{n_{L}+n_{R}}\sum_{\mu \wedge \upsilon }1-{\text{cos}}^{2}\;\theta_{\mu \wedge \upsilon }\\ \;+\frac{\alpha }{n_{N}n_{S}}\sum_{k,l}{\left(\text{tan}\;\theta_{k}+\text{tan}\;\theta_{l}\right)}^{2} \end{array}\;,$$

$$c_{12}=\frac{1-\alpha }{n_{N}+n_{S}}\sum_{k\wedge l}\text{cos}\;\theta_{k\wedge l}\;\text{sin}\;\theta_{k\wedge l}+\frac{1-\alpha }{n_{W}+n_{E}}\sum_{\mu \wedge \upsilon }\text{cos}\;\theta_{\mu \wedge \upsilon }\;\text{sin}\;\theta_{\mu \wedge \upsilon }\;,$$

$$c_{13}=-\frac{1-\alpha }{n_{W}+n_{E}}\sum_{\mu \wedge \upsilon }x_{\mu \wedge \upsilon }-x_{\mu \wedge \upsilon }\;{\text{cos}}^{2}\;\theta_{\mu \wedge \upsilon }\;,$$

$$c_{14}=\frac{1-\alpha }{n_{N}+n_{S}}\sum_{k\wedge l}y_{k\wedge l}\;\text{cos}\;\theta_{k\wedge l}\;\text{sin}\;\theta_{k\wedge l}+L\frac{\alpha }{n_{N}n_{S}}\sum_{k,l}\text{tan}\;\theta_{k}+\text{tan}\;\theta_{l}\;,$$

$$\begin{array}{l} c_{22}=\frac{1-\alpha }{n_{N}+n_{S}}\sum_{k\wedge l}1-{\text{sin}}^{2}\;\theta_{k\wedge l}+\frac{1-\alpha }{n_{W}+n_{E}}\sum_{\mu \wedge \nu }1-{\text{sin}}^{2}\;\theta_{\mu \wedge \nu }\\ \;+\frac{\alpha }{n_{N}n_{S}}\sum_{k,l}{\left(\text{tan}\;\theta_{k}+\text{tan}\;\theta_{l}\right)}^{2} \end{array}\;,$$

$$c_{23}=\frac{1-\alpha }{n_{W}+n_{E}}\sum_{\mu \wedge \nu }x_{\mu \wedge \nu }\;\text{cos}\;\theta_{\mu \wedge \nu }\;\text{sin}\;\theta_{\mu \wedge \nu }+W\frac{\alpha }{n_{N}n_{S}}\sum_{k,l}\text{tan}\;\theta_{k}+\text{tan}\;\theta_{l}\;,$$

$$c_{24}=-\frac{1-\alpha }{n_{N}+n_{S}}\sum_{k\wedge l}y_{k\wedge l}-y_{k\wedge l}\;{\text{sin}}^{2}\;\theta_{k\wedge l}\;,$$

$$c_{33}=\alpha \;W^{2}+\frac{1-\alpha }{n_{W}+n_{E}}\sum_{\mu \wedge \nu }x_{\mu \wedge \nu }^{2}-x_{\mu \wedge \nu }^{2}\;{\text{cos}}^{2}\;\theta_{\mu \wedge \nu }\;,\;c_{34}=0$$

$$c_{44}=\alpha \;L^{2}+\frac{1-\alpha }{n_{N}+n_{S}}\sum_{k\wedge l}y_{k\wedge l}^{2}-y_{k\wedge l}^{2}\;{\text{sin}}^{2}\;\theta_{k\wedge l}\;,$$

$$c_{21}=c_{12},\;c_{31}=c_{13},\;c_{41}=c_{14},\;c_{32}=c_{23},\;c_{42}=c_{24},\;c_{34}=c_{43},$$

$$\begin{array}{l} d_{1}=\frac{1-\alpha }{n_{N}+n_{S}}\sum_{k\wedge l}x_{k\wedge l}-x_{k\wedge l}{\;\text{cos}}^{2}\;\theta_{k\wedge l}-\frac{1-\alpha }{n_{W}+n_{E}}\sum_{\mu \wedge \nu }y_{\mu \wedge \nu }\;\text{cos}\;\theta_{\mu \wedge \nu }\;\text{sin}\;\theta_{\mu \wedge \tau }\\ \;+\frac{\alpha }{n_{W}n_{E}}\sum_{k,l}\left(x_{k}\;\text{tan}\;\theta_{k}+x_{l}\;\text{tan}\;\theta_{l}\right)\left(\text{tan}\;\theta_{k}+\text{tan}\;\theta_{l}\right) \end{array}\;,$$

$$\begin{array}{l} d_{2}=\frac{1-\alpha }{n_{W}+n_{E}}\sum_{\mu \wedge \nu }y_{\mu \wedge \nu }-y_{\mu \wedge \nu }\;{\text{sin}}^{2}\;\theta_{\mu \wedge \tau }-\frac{1-\alpha }{n_{N}+n_{S}}\sum_{k\wedge l}x_{k\wedge l}\;\text{cos}\;\theta_{k\wedge l}\;\text{sin}\;\theta_{k\wedge l}\\ \;+\frac{\alpha }{n_{W}n_{E}}\sum_{\mu ,\upsilon }\left(y_{\mu }\;\text{tan}\;\theta_{\mu }+y_{\nu }\;\text{tan}\;\theta_{\nu }\right)\left(\text{tan}\;\theta_{\mu }+\text{tan}\;\theta_{\nu }\right) \end{array}\;,$$

$$\begin{array}{l} d_{3}=\frac{1-\alpha }{n_{W}+n_{E}}\sum_{\mu \wedge \nu }x_{\mu \wedge \nu }y_{\mu \wedge \nu }\;\text{cos}\;\theta_{\mu \wedge \nu }\;\text{sin}\;\theta_{\mu \wedge \tau }\\ \;+W\frac{\alpha }{n_{W}n_{E}}\sum_{\mu ,\upsilon }y_{\mu }\;\text{tan}\;\theta_{\mu }+y_{\nu }\;\text{tan}\;\theta_{\nu } \end{array}\;,$$

$$d_{4}=\frac{1-\alpha }{n_{N}+n_{S}}\sum_{k\wedge l}x_{k\wedge l}y_{k\wedge l}\;\text{cos}\;\theta_{k\wedge l}\;\text{sin}\;\theta_{k\wedge l}+L\frac{\alpha }{n_{N}n_{S}}\sum_{k,l}x_{k}\;\text{tan}\;\theta_{k}+x_{l}\;\text{tan}\;\theta_{l}\;.$$

The matrix *C* is symmetric and, therefore, its inverse exists, except in degenerated cases. To get the estimate of the location, we rescale the x-coordinate and y-coordinate using their respective compression factors. Thus, we get the final estimate for the location of self as:

$$\left(\begin{array}{c} {\tilde{x}}_{s}\\ {\tilde{y}}_{s} \end{array}\right)=\left(\begin{array}{c} {\hat{x}}_{s}\;/\;\hat{\eta }\\ {\hat{y}}_{s}\;/\;\hat{\xi } \end{array}\right).$$ (14)

### Oscillatory interference model for grid cell firing

To represent the pattern of grid cell firing based on location estimates from either the moving feature system or the static feature system, we used a simple model of grid cell firing. Note that the result of our simulations does not depend upon the use of a specific grid cell model. In fact, we also demonstrate similar results using the pattern of grid cell firing generated with an attractor model of grid cells (S1 Fig and S2 Fig). The oscillatory interference model was primarily chosen for simplicity of implementation. We used the oscillatory interference model initially proposed by Burgess [6, 16] and subsequently used by others [17, 40]. For this formulation of the oscillatory interference model, we take the 2D location signal ${\vec{x}}_{j}$ from either Eq 9 or Eq 14 to model the firing of grid cells in the dorsal or ventral part of the entorhinal cortex, respectively. The oscillatory interference model uses oscillations based on different heading directions in the environment. In the implementation used here, we used a location input instead of a velocity input. This location signal is projected onto a “basis system” of three vectors ${\vec{b}}_{k}$, which are arranged at 120 deg angular difference. We have ${\vec{b}}_{1}={\left(\text{cos}\;0{}^{\circ},\;\text{sin}\;0{}^{\circ}\right)}^{t}$, ${\vec{b}}_{2}={\left(\text{cos}\;120{}^{\circ},\;\text{sin}\;120{}^{\circ}\right)}^{t}$, and ${\vec{b}}_{3}={\left(\text{cos}\;240{}^{\circ},\;\text{sin}\;240{}^{\circ}\right)}^{t}$. The result of this projection is modulated by the angular frequency *ω* and the parameter *β*_*l*_, which determines the grid spacing. The resulting signal is added to a time dependent modulation *ωt* to generate the phase of three different oscillations corresponding to the different directions above. This is the argument for three oscillations, which are combined with another baseline oscillation that simply has *ωt* as argument. These four oscillations form an interference pattern in the 2D plane and for this reason the model is called the oscillatory interference model. The model equation is [16]:

$$spike(t_{lj})=\{\begin{array}{cc} 1 & \left(\prod_{k=1}^{3}\text{cos}\left(\omega \;t_{j}\right)+\text{cos}\left(\omega \;t+\omega \;\beta_{l}\;{\vec{x}}_{j}\cdot {\vec{b}}_{k}\right)\right)>\Theta \\ 0 & otherwise \end{array}.$$ (15)

The biological interpretation of this model Eq 15 can take on different forms. For example, there may be a network oscillation with phase *ωt* and separate, individual local oscillations with phases $\omega (t+\beta_{l}\;{\vec{x}}_{j}\cdot {\vec{b}}_{k})$, which are the oscillations modulated by the location signal ${\vec{x}}_{j}$ for each basis vector and by the parameter *β*_*l*_. The oscillation *ω* could be provided by the theta rhythm oscillations regulated by the medial septum [36]. In the model interpretation the three oscillations indexed by *k* interfere at an individual grid cell. Each oscillation is associated with a direction in the 2D plane. These oscillations lead to a band of firing with an inter-band distance of 1/(*β f*), because the constructive interference between a purely somatic oscillation *f* = *ω*/(2*π*) and dendritic oscillation *f* + *f β* results in an oscillation with the overall envelope of frequency *f* + *f β–f* = *f β*. The multiplicative or additive combination of all three bands reaches values above the threshold *Θ* in vertices of a hexagonal grid given the 120 deg difference between basis vectors. The grid spacing is $G_{l}=2/(\sqrt{3}\beta_{l}f)$ and differs for the dorsal or ventral part of the entorhinal cortex. We model this by choosing two values for the parameter *β*_*l*_ (see Table 1).

### Attractor model for grid cell firing

The attractor model simulates the regular hexagonal firing pattern of grid cells using a previously described model [18]. In this previously published model, a population of *n* = *n*_*x*_ × *n*_*y*_ cells are arranged as a 2D array with connections at the boundaries using a twisted torus topology. The twisted property of the topology is necessary to produce the hexagonal distribution of grid cell firing in contrast to a square array that would occur without the twisting. We report firing for the cell with the linear index *n*_*x*_×*n*_*y*_ *—n*_*y*_/2. For our model simulations we set *n*_*x*_ = 10 and *n*_*y*_ = 9. Locations of cells on the torus are ${\vec{c}}_{k}={\left(x_{i},y_{j}\right)}^{t}$ with coordinates *x*_*i*_ = (*i* − 0.5)/*n*_*x*_ and $y_{j}=\sqrt{3}/2(j-0.5)/n_{y}$ for the indices *i* = 1…*n*_*x*_ and *j* = 1…*n*_*y*_. Note that *k* is the linear index of *i* and *j*, e.g. *k* = *j*+*i*×*n*_*y*_. The activity of all cells is represented in the vector $\vec{a}(t)$. Here this vector has 90 components. Synaptic weights between cells are defined through the matrix *W* with the entries

$$w_{kl}(t)=I\;\text{exp}(-\frac{{\left\|{\vec{c}}_{k}-{\vec{c}}_{l}+\alpha \;R_{\gamma }{\vec{v}}_{2D}(t)\right\|}_{tri}^{2}}{\sigma^{2}})-T\;\text{with}\;k=1\ldots \text{n}\;\text{and}\;l=1\ldots \text{n}.$$ (16)

The weights are defined using the 2D velocity signal ${\vec{v}}_{2D}(t)$. In the case of the static feature system, we calculate the temporal difference of the location signal to define this velocity. The norm ‖⋅‖_*tri*_ in Eq 16 implements a distance measure on the twisted torus topology. The parameter *I* = 0.3 models the peak synaptic strength. The parameter *T* = 0.05 introduces inhibition for weights at the tail end of the distribution. The parameter *α* controls the input gain of the velocity and the grid spacing is approximately 1.02 − 0.48 log_2_(*α*). For a simulated dorsal grid cell we set the gain *α*_*1*_ = 1.4×10^−3^ and for a ventral grid cell we set the gain *α*_*1*_ = 0.9×10^−3^. The matrix *R*_*γ*_ rotates the grid orientation, here, we set *γ* = 0°. The standard deviation *σ* determines the size of the firing fields, here *σ* = 0.24 meters. The activities $\vec{a}(t)$ are updated through:

$$\vec{b}(t+1)=W(t)\vec{a}(t)\;\text{and}$$ (17a)

$$\vec{a}(t+1)={\left[(1-\tau )\;\vec{b}(t+1)+\tau \;\vec{b}(t)/\sum_{i=1}^{n}{\vec{a}}_{i}(t)\right]}^{+}$$ (17b)

Eq 17a models the interaction between cells using the synaptic weight matrix *W*(t). The second Eq 17b computes an exponentially weighted average. The factor 1-*τ* weighs the history of activation against the current activation, which has the weight *τ*, here *τ* = 0.8. In addition, Eq 17b normalizes the activations against the sum of all activities. All activations are positive due to the applied half-wave rectification [⋅]^+^. Each activity $\vec{a}$ is initialized randomly using a uniform distribution that ranges between 0 and (√*N*)^-1^. A model cell spikes if its activity is above the threshold level of *η* = 0.1. Formally, we express this as:

$${spike}_{k}(t)=\{\begin{array}{ll} 1 & a_{k}(t)>\eta \\ 0 & otherwise \end{array}\;\text{for}\;k=1\ldots n.$$ (18)

As shown in the results section, the properties of selective compression of grid cell firing field spacing due to shifts of barriers are the same for this attractor model as they were for the oscillatory interference model.

### Noise models

We use two methods to introduce noise in our simulations and to study the robustness of our proposed methods for the estimation of location. The first method superimposes independent and identically distributed Gaussian noise onto angular velocities of feature locations

$$\left(\begin{array}{c} {\dot{\theta }}_{n}\\ {\dot{\phi }}_{n} \end{array}\right)=\left(\begin{array}{c} \dot{\theta }\\ \dot{\phi } \end{array}\right)+\left(\begin{array}{c} \dot{\vartheta }\\ \dot{\xi } \end{array}\right)$$ (19)

with $\dot{\vartheta }$ and $\dot{\xi }$ ϵ *N*(*μ*_*v*_, *σ*_*v*_), where *N*(*μ*_*v*_, *σ*_*v*_) denotes the normal distribution with mean *μ*_*v*_ and standard deviation *σ*_*v*_. Notice that $\dot{\vartheta }$ and $\dot{\xi }$ are drawn for each time step. The sequence of drawn variables $\dot{\vartheta }$ and $\dot{\xi }$ is temporally uncorrelated, assuming a random number generator of infinite sequence. In practice the sequence is finite but larger than the number of samples that we drew.

The second method superimposes noise onto the observed angular direction of landmarks

$$\left(\begin{array}{c} \theta_{n}\\ \phi_{n} \end{array}\right)=\left(\begin{array}{c} \theta \\ \phi \end{array}\right)+\left(\begin{array}{c} \vartheta \\ \xi \end{array}\right)$$ (20)

with *ϑ* and *ξ* ϵ *N*(*μ*_*l*_, *σ*_*l*_), where *N*(*μ*_*l*_, *σ*_*l*_) denotes the normal distribution with mean *μ*_*l*_ and standard deviation *σ*_*l*_. As before, the sequence of drawn *ϑ* and *ξ* is uncorrelated.

Obviously, the parameters of normal distribution used in these two methods cannot be directly related to each other, one set of random variables $\dot{\vartheta }$ and $\dot{\xi }$ is applied in the domain of image velocities and another set of random variables *ϑ* and *ξ* is applied in the angular domain. As measure of comparison we chose the domain-independent signal to noise ratio in decibels:

$$SNR[dB]=20\;{\text{log}}_{10}\frac{\sum_{i=1}^{k}s_{i}^{2}}{\sum_{i=1}^{k}{\left(m_{i}-s_{i}\right)}^{2}}$$ (21)

with *k* samples of the noise-free signal *s* and the measured signal *m*, which includes the noise. For the angular velocities our estimate is only based on the azimuthal angular velocity and, thus, $s_{i}={\dot{\theta }}_{i}$ and $m_{i}={\dot{\theta }}_{i,n}$. For the angles of landmarks our estimate uses both azimuth and elevation and, we define the noise-free signal by *s*_*i*_ = *θ*_*i*_ for the first *k* samples and *s*_*i*_ = *ϕ*_*i*_ for another *k* samples. Analogously, we define the measured signal by *m*_*i*_ = *θ*_*i*,*n*_ for the first *k* samples and *m*_*i*_ = *ϕ*_*i*,*n*_ for another k samples. Thus, the sums in Eq 21 range over 2*k*, respectively. This noise characterization allows for comparison of the strength of noise in the two domains of angular velocities and angles.

## Supporting Information

S1 Fig Shows the simulation results for the compression of a box using the attractor model. (Guanella et al., 2007). (A) Grid cell firing pattern (grid score 1.70) from a simulated dorsal entorhinal cell for configuration A and (B) for configuration B (grid score 1.63). (C) Correlation value r^2^ for compression of B relative to A shows peak value at ≈ -6.8% change. (D) Firing pattern (grid score 1.27) from the simulated ventral entorhinal neuron for configuration A and (E) configuration B (grid score -1.41). (F) Correlation value r^2^ for compression of B relative to A shows that B needs to be increased by ≈ 98.8% to obtain maximum correlation with A. These results are qualitatively the same as for the velocity controlled oscillator model (Fig 3). For the simulation of the attractor model, we used the parameters for grid scale *α*_*1*_ = 1.4e-3 (dorsal grid cell), *α*_*2*_ = 0.9e-4 (ventral grid cell), for grid orientation *β* = 0 degrees, for number of cells *N*_*x*_ = 9 and *N*_*y*_ = 10, for the stabilization strength *τ* = 0.8, the intensity *I* = 0.8, the standard deviation of the Gaussian *σ* = 0.24 meters, the shift parameter *T* = 0.05, and the threshold for spiking of 0.1 (see also Table 1).
(TIF)

S2 Fig Compares the grid cell firing patterns for the velocity controlled oscillator model and the attractor model for noise in the observed visual angles of landmarks or in the image velocities of the optic flow using Gaussian noise with and without zero-mean. The firing patterns from the velocity controlled oscillator model are shown in (A) for bias-free noise and the static feature system (grid score 1.84) in (B) for bias-free noise and the moving feature system (grid score 1.67), in (C) for biased noise and the static feature system (grid score -1.29), and in (D) for biased noise and the static feature system (grid score 1.66). Similarly, the firing patterns from the attractor model are shown in (E) for bias-free noise and the static feature system (grid score 1.50) in (F) for bias-free noise and the moving feature system (grid score 1.61), in (G) for biased noise and the static feature system (grid score -0.75), and in (H) for biased noise and the static feature system (grid score 1.56). The simulation for the two grid models are qualitatively the same, comparing the first with the second row.
(TIF)

S1 File Contains Matlab code to replicate the simulation results of this article. (ZIP)
