# Supplementary figures and images for: Differences in Visual-Spatial Input May Underlie Different Compression Properties of Firing Fields for Grid Cell Modules in Medial Entorhinal Cortex
Source: PLoS Comput Biol. 2015 Nov 19;11(11):e1004596. doi: 10.1371/journal.pcbi.1004596 (PMC4652908; doi:10.1371/journal.pcbi.1004596)

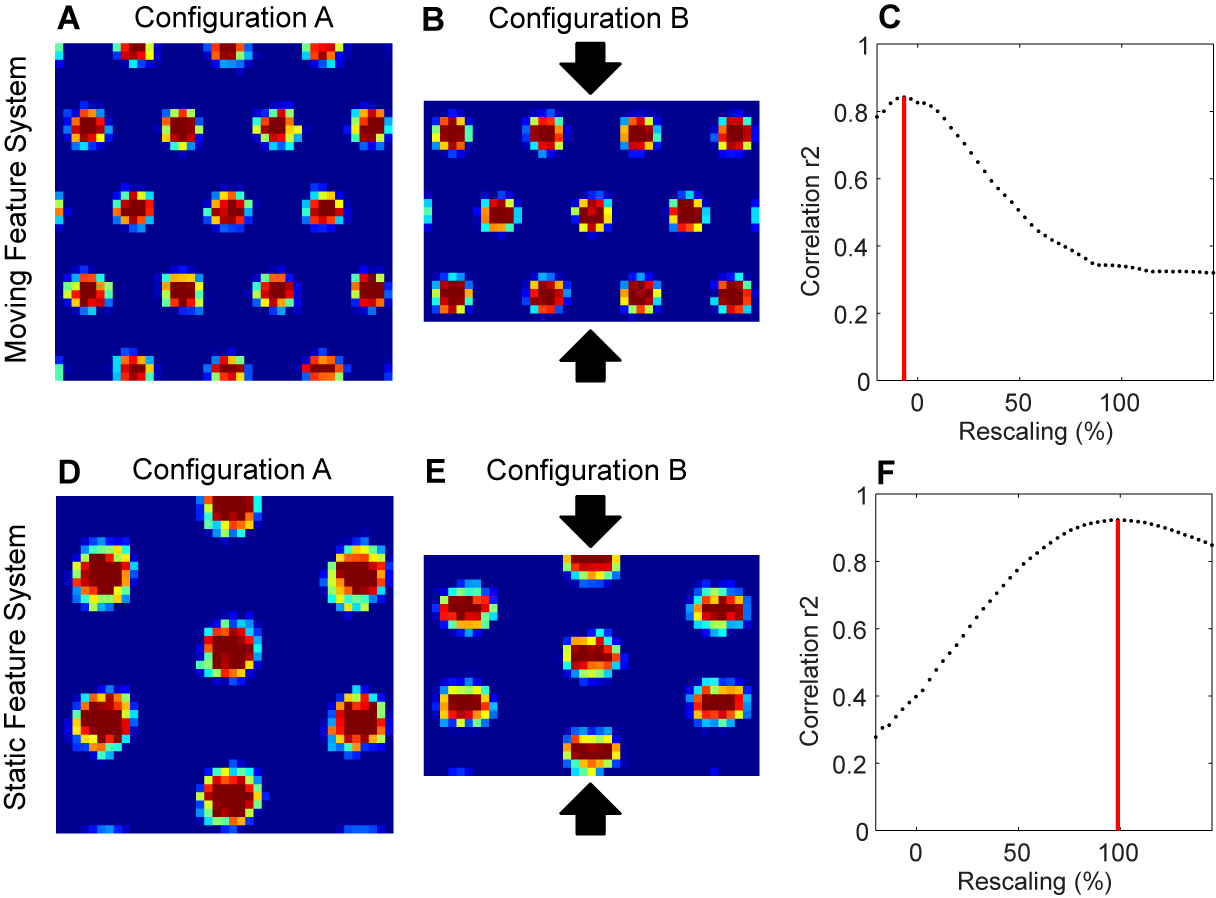

Supplement: S1 Fig — (Guanella et al., 2007). (A) Grid cell firing pattern (grid score 1.70) from a simulated dorsal entorhinal cell for configuration A and (B) for configuration B (grid score 1.63). (C) Correlation value r2 for compression of B relative to A shows peak value at ≈ -6.8% change. (D) Firing pattern (grid score 1.27) from the simulated ventral entorhinal neuron for configuration A and (E) configuration B (grid score -1.41). (F) Correlation value r2 for compression of B relative to A shows that B needs to be increased by ≈ 98.8% to obtain maximum correlation with A. These results are qualitatively the same as for the velocity controlled oscillator model (Fig 3). For the simulation of the attractor model, we used the parameters for grid scale α 1 = 1.4e-3 (dorsal grid cell), α 2 = 0.9e-4 (ventral grid cell), for grid orientation β = 0 degrees, for number of cells N x = 9 and N y = 10, for the stabilization strength τ = 0.8, the intensity I = 0.8, the standard deviation of the Gaussian σ = 0.24 meters, the shift parameter T = 0.05, and the threshold for spiking of 0.1 (see also Table 1). (TIF) [file pcbi.1004596.s001.tif]

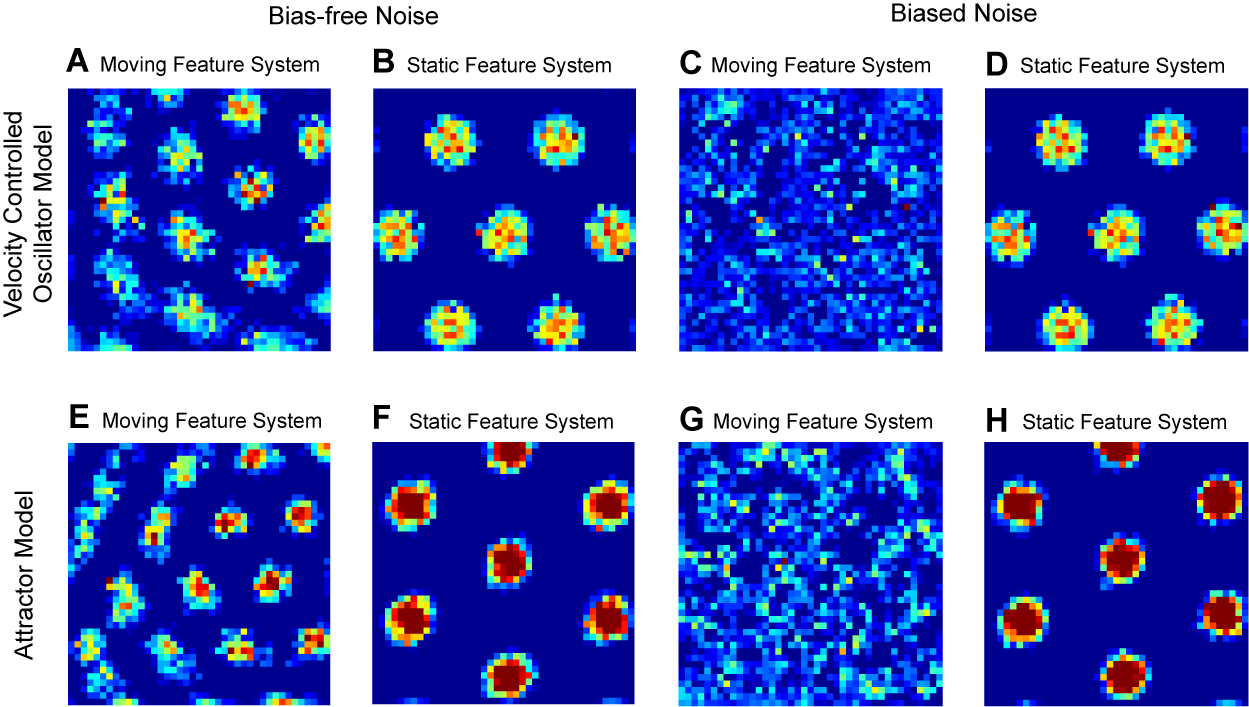

Supplement: S2 Fig — The firing patterns from the velocity controlled oscillator model are shown in (A) for bias-free noise and the static feature system (grid score 1.84) in (B) for bias-free noise and the moving feature system (grid score 1.67), in (C) for biased noise and the static feature system (grid score -1.29), and in (D) for biased noise and the static feature system (grid score 1.66). Similarly, the firing patterns from the attractor model are shown in (E) for bias-free noise and the static feature system (grid score 1.50) in (F) for bias-free noise and the moving feature system (grid score 1.61), in (G) for biased noise and the static feature system (grid score -0.75), and in (H) for biased noise and the static feature system (grid score 1.56). The simulation for the two grid models are qualitatively the same, comparing the first with the second row. (TIF) [file pcbi.1004596.s002.tif]
